# Relational information framework, causality, unification of quantum interpretations and return to realism through non-ergodicity

**DOI:** 10.1038/s41598-025-90225-7

**Published:** 2025-03-10

**Authors:** Oded Shor, Felix Benninger, Andrei Khrennikov

**Affiliations:** 1 Felsenstein Medical Research Centre, Petach Tikva, Israel; 2 Department of Neurology, Rabin Medical Centre, Petach Tikva, Israel; 3 Faculty of Medicine, Tel Aviv University, Tel Aviv, Israel; 4 Department of Mathematics, Faculty of Technology, Linnaeus University, Vaxjö, Sweden

**Keywords:** p-Adic numbers, Dendrograms, Relational information, Bohmian mechanics, Minkowski-like parameter space, Many worlds interpretation, Non-ergodicity, Applied mathematics, Information theory and computation, Quantum information, Theoretical physics, Statistical physics

## Abstract

In the framework of relational information, we explore analogs of physical theories and their properties. Specifically, we investigate the causal characteristics of relational information, examining how initial knowledge impacts future relational understanding of the universe/system. To achieve this, we establish a parameter space defining relational structures called dendrograms, exhibiting causal properties akin to those of Minkowski metric. Subsequently, we propose a statistical-dynamical model on this Minkowski-like parameter space, unifying Bohmian and Many Worlds interpretations of quantum theory in the framework of relational information. Additionally, we provide an analytical proof of the non-ergodicity of the relational information framework, revealing CHSH inequality violations as an emergent phenomenon. Our focus on relational information underscores its significance across scientific disciplines, where a single measurement or observation lacks meaning without context.

**Supplementary Information:**

The online version contains supplementary material available at 10.1038/s41598-025-90225-7.

## Introduction

Dendrogramic Holographic Theory (DHT) is a singular postulate theory of relational information, conceived through a series of studies^1–6^. This theory represents a relational-event-observational approach to physics and the broader realm of natural science^7,8^. The foundational postulate underpinning and driving all theoretical implications is the adherence to Leibniz’s Principle^9^ and its empiricist epistemic reinterpretation which resembles Mach’s interpertation: “If we are unable to distinguish two states of things from each other by any scientific means, then science ought to regard them as identical and take no notice of the difference”^10^.

Leibniz’s Principle, also known as the Principle of the Identity of Indiscernibles, is typically expressed in a specific manner:

If, for every property $\text{F}$, object $\text{x}$ possesses $\text{F}$ if and only if object $\text{y}$ possesses $\text{F}$, then $\text{x}$ is identical to $\text{y}$. In symbolic logic notation, this can be represented as $\forall F(Fx \leftrightarrow Fy) \to x = y$. In simpler terms, if $\text{x}$ and $\text{y}$ are distinct entities, there must be at least one property that distinguishes them, ensuring their non-identity.

This principle is an ontic tool for the relational metaphysics (orignaly served for distinguishing between monads^9^).

The epistemic view of DHT pertains to relational information concerning events as measured by an observer. In this context, measurement/observation about a single event lacks significance and cannot exist in isolation; its meaning derives only from its comparison to information obtained from other events. According to the epistemically refined Leibniz’s principle which corresponds to Mach’s view, if a particular observer cannot distinguish any features that differentiate two observations or measurements of distinct events, then these two events are considered identical to that observer. In subsequent sections we will demonstrate the linkage between such epistemic principle to the original ontic principle. We have to note that with that within the more pragmatic Epistemic view the reinterpretations given to the original ontic Leibnitz principle came with the cost of added postulates like the existance of observers and measurements (or “sensations” according to Mach).

We will call throughout this study the epistemic reinterpretation of the ontic Leibnitz principle – Leibnitz principle or epistemic Leibnitz principle in cases we will refer to the original Leibnitz principle we will refer to it as the ontic Leibnitz principle. In the current study our models will be derived mainly from an epistemic view but eventually (or in the limit of infinity) will be well connected to an ontic model. The epistemic-ontic connection will be exemplified for each of the results we will derive below.

### P-adic treelike formalization of Leibniz’s principle

DHT relies on p-adic topology which is governed by the p-adic ultrametric and draws relational information from p-adic tree structures called dendrograms. To distinguish observations, an observer uses inquiries often yes/no questions, depicted as dendrograms resembling decision trees. These trees quantify observations relationships based on divergent answers.

In the limit of infinite events, their inherent nature is represented as an infinite p-adic tree, characterized by homogeneity and vertices with *p* > 1 edges. These trees possess an algebraic structure and topology corresponding to this configuration^11^.

In the p-adic topology, triangles are always isosceles due to the p-adic ultrametric satisfying the strong triangle inequality. Here, the distance between two branches in the tree depends on their shared root (indicating more questions answered similarly), with a longer shared root implying a shorter distance. Additionally, “open” and “closed” balls are defined as follows:

$${B}_{-}\left(R;a\right)=\left\{x :{r}_{p}\left(a,x\right)<R\right\}, B\left(R;a\right)=\left\{x :{r}_{p}\left(a,x\right)\le R\right\}$$

$$\text{W}\text{h}\text{e}\text{r}\text{e}: {r}_{p}\left(a,x\right)=|a-x{|}_{p} \text{ i}\text{s} \text{ t}\text{h}\text{e} \text{ p}-\text{a}\text{d}\text{i}\text{c} \text{ d}\text{i}\text{s}\text{t}\text{a}\text{n}\text{c}\text{e} \text{ b}\text{e}\text{t}\text{w}\text{e}\text{e}\text{n} \text{ t}\text{h}\text{e} \text{ p}\text{o}\text{i}\text{n}\text{t}\text{s} \text{ a} \text{a}\text{n}\text{d} \text{ x}$$

Since branches symbolize distinct events relations to all other events, the relational event space, whether finite or infinite in nature, is furnished with a p-adic ultrametric. This connection between DHT and p-adic analysis intertwines with the realms of theoretical physics^12–27^. This relationship includes investigations by Parisi et al.^26^ into complex disordered systems within the p-adic framework and the broader ultrametric context, as expounded in article^27^. General trees, in general, feature an ultrametric topology, and such topological spaces have found extensive utility in the theory of complex disordered systems^16,26^.

The Leibniz principle necessitates event differentiation through a question-based process, represented in the p-adic number field. This process, forming a p-adic tree, associates each event with all others. Thus, Leibniz’s principle leads to Machian relationism, inherently arising from the p-adic tree representation of events. Embracing this principle yields a background-independent theory, akin to shape dynamics, loop quantum gravity, spin foams, and causal set theory^28–32^. These two outcomes are not merely assumed, as seen in theories like shape dynamics and Brans-Dicke theory, but rather emerge intrinsically from the structure of the p-adic tree^28,29,32^.

Recently, theoretical developments^33–37^ showed the emergence of our known physical theories from machine learning perspectives. In these studies, the laws of physics are shown to emerge as a learning procedures. For instance, action principle effectively determines the “best” or “most efficient” pathway a system follows according to certain physical laws. much like an optimization process an action principle can be seen as a mechanism by which the system “learns” the optimal path under constraints, In machine learning, we typically define a loss function that quantifies how far a system’s predictions or behaviors are from desired outcomes. The learning algorithm then iteratively updates parameters to minimize this loss, effectively “learning” the best parameter values for the given problem. In essence, both systems “converge” toward an optimal configuration—whether a physical path (action principle) or a set of model parameters (learning algorithm)—guided by an optimization process. Thus, action principles might be referred trivially as a particular learning procedure^33–37^. In recent studies^5,6^, we have already drawn attention to intriguing parallels between our approach, which yields the emergence of quantum theory from DHT, and the neural network model of the universe as described in articles^33–37^. Notably, we should highlight that p-adic neural networks have been investigated in conjunction with Euclidean quantum field theory, as detailed in references^36,37^, thereby establishing a closer connection between our approach and the domain of machine learning.

We follow a structured approach to establish a relational framework, specifically in the form of a dendrogram. This dendrogram exhibits a distinct branching pattern through a p-adic expansion. The dendrogram is represented by p-adic (in this study we use mainly 2-adic in our numerical simulations) numbers, each depicting an event’s relationship with other events as observed by the observers. It requires a minimum of two events to construct a dendrogram due to its relational nature.

**Fig. 1:**
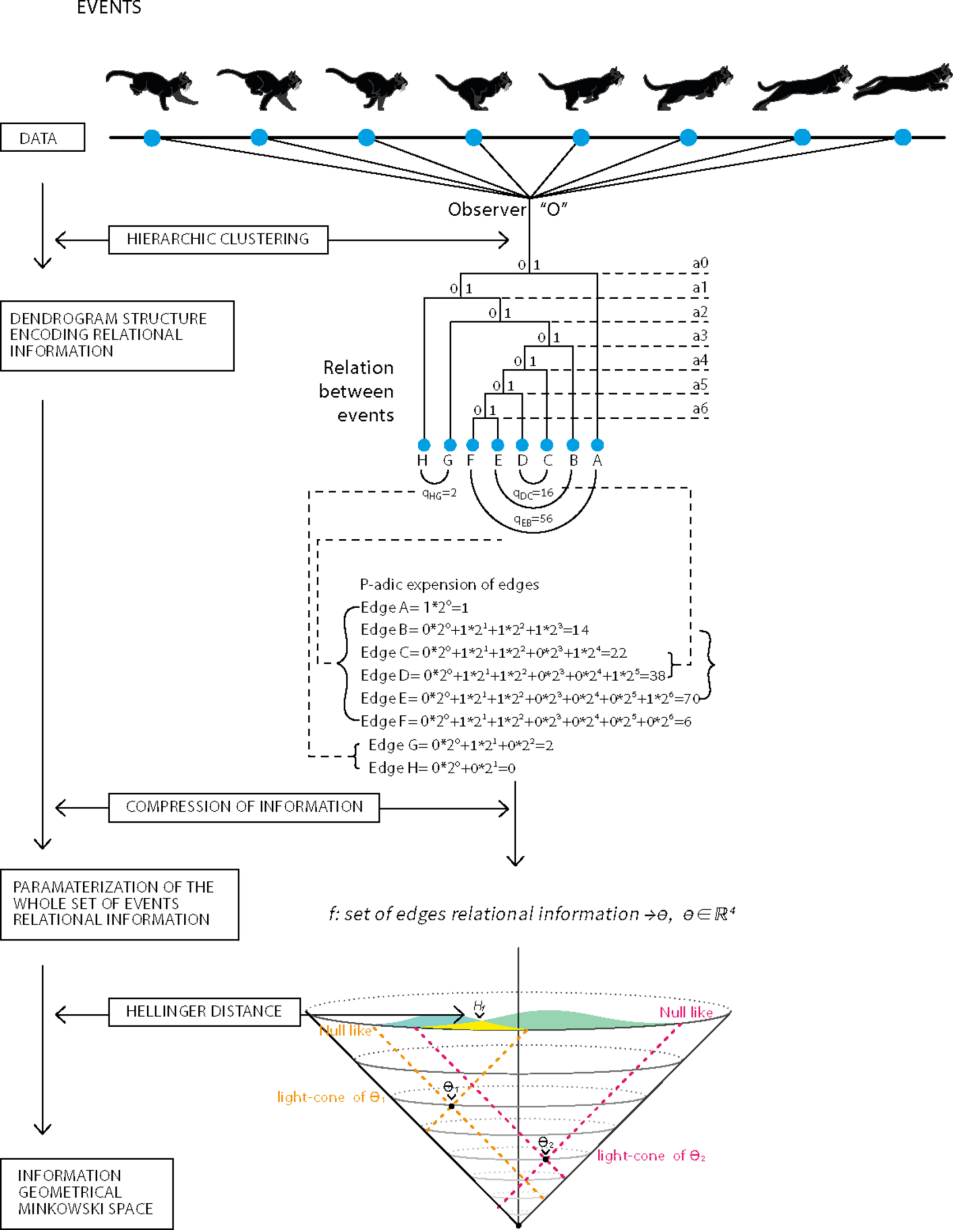
Illustration of the relational information framework developed in this study. Data events are collected by an observer, and pairwise distances between events are calculated to apply Hierarchical clustering, resulting in a dendrogramic tree. Each event in the tree is represented by a binary string or p-adic expansion. Numerical examples demonstrate calculations and 2-adic differences. The set of these strings/p-adic expansions constitutes the relational information between all collected data events, describing the dendrogram’s structure. We compress this relational information set into a 4D parameter space without information loss. Utilizing the information.

A dendrogram essentially takes the shape of a finite tree, serving as an observer’s epistemic representation of reality (which is subjective in nature, see Supplementary Appendix Section A1). These finite trees are constructed, epistemically, according to four steps: data collection, hierarchical clustering of the data, production of agglomerative cluster tree and the dendrogram representation of relations where a longer shared path of the dendrogram signifies a closer relationship between events based on chosen metrics (Fig. 1).

This study aims to investigate relational information analogs of physical theories and their causal characteristics. We seek to prove the existence of a parameter space defining relational information structures called dendrograms, with causal properties akin to the Minkowski metric (Fig. 1). Additionally, we will present a statistical-dynamical model on this parameter space to unify interpretations of quantum theory. We also aim to provide an analytical proof for the non-ergodicity of relational information, that might explain CHSH inequality violations. Our focus on relational information suggests broad applicability across scientific domains.

### Dendrogram relational structure as a configuration of nodes

**Fig. 2:**
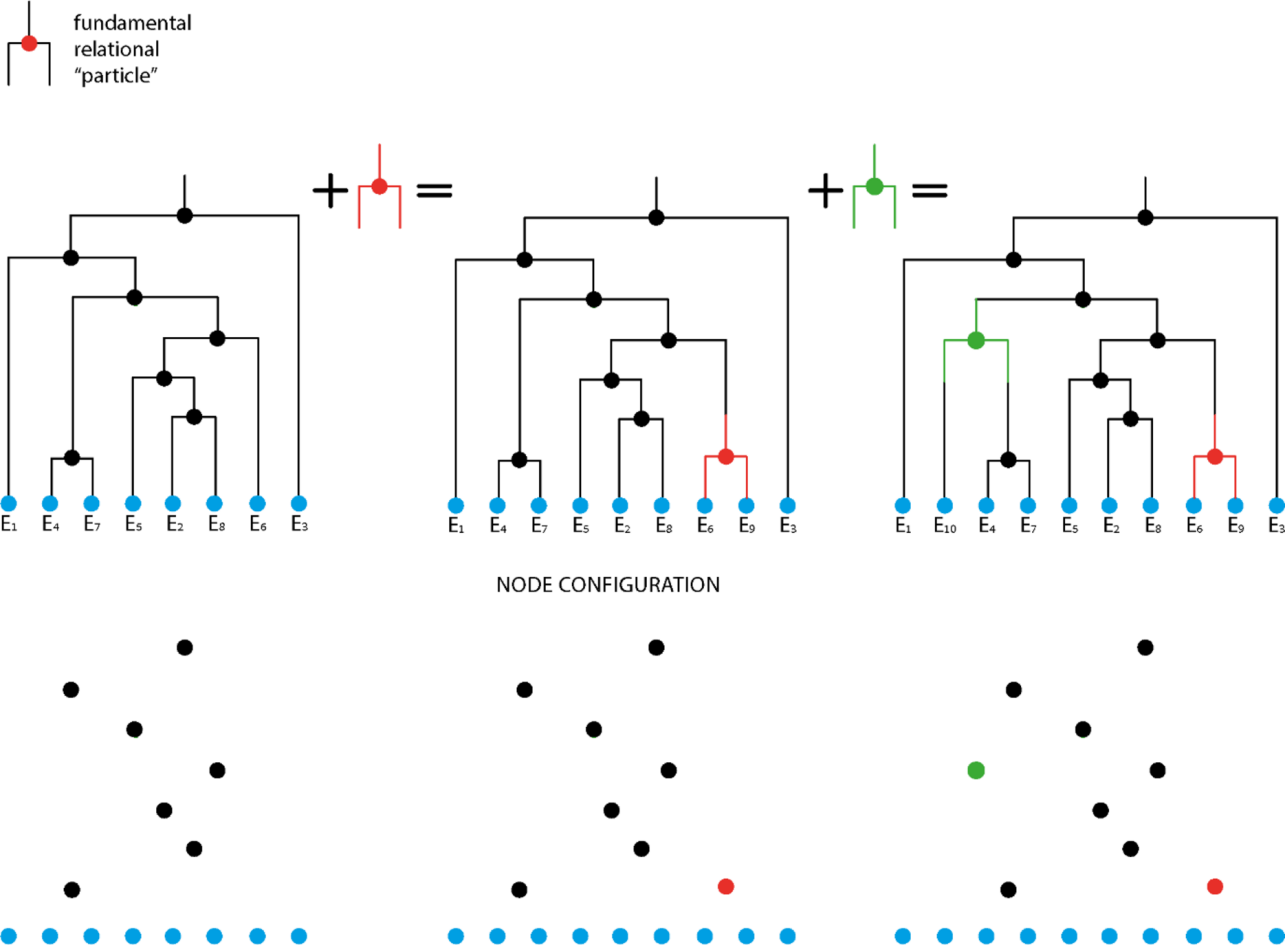
The fundamental “question” (fundamental relational “particle”). Example of consecutive evolvement of the relational information dendrogramic structure by adding one “questions” in each step (upper row). At the lower the configuration of these questions in the p-adic field is presented upon same addition of “question”. Each such configuration can be constructed from a union of unique partial configurations.

The dendrogram’s structure resembles a decision-making tree, with branching nodes representing fundamental “question” (fundamental relational “particle”). These nodes are positioned in the p-adic field based on their p-adic expansion. Using p-adic numbers, any dendrogram can be constructed, where leaf nodes represent non-branching nodes (Fig. 2).

Additionally, dendrograms can be built from entities less fundamental than the “question” by combining partial configurations of all questions. A sequence of events can be seen as a union of unique partial “question” configurations. These configurations can be mapped to a lower level dendrogram, resulting in indistinguishability, as observed in previous numerical simulations^1–4^.

geometry Hellinger metric, we demonstrate causal relations in the parameter space, aligning with those found in Minkowski space of special relativity.

## Causal structure on the space of dendrograms

In our model, we introduce $\text{O}{_i}\text{ where }\text{i}=\text{1,2},\dots . N\to {\infty }$ observers who collects/measures events/data. Thus, an observer with already m events collected e_1_, e_2_… e_m_ will measure each time a single event, e_m+1_. Each time an observer O_i_ measures an event, he constructs a dendrogram using the procedure outlined in the Introduction and Supplementary Appendix.

**Fig. 3:**
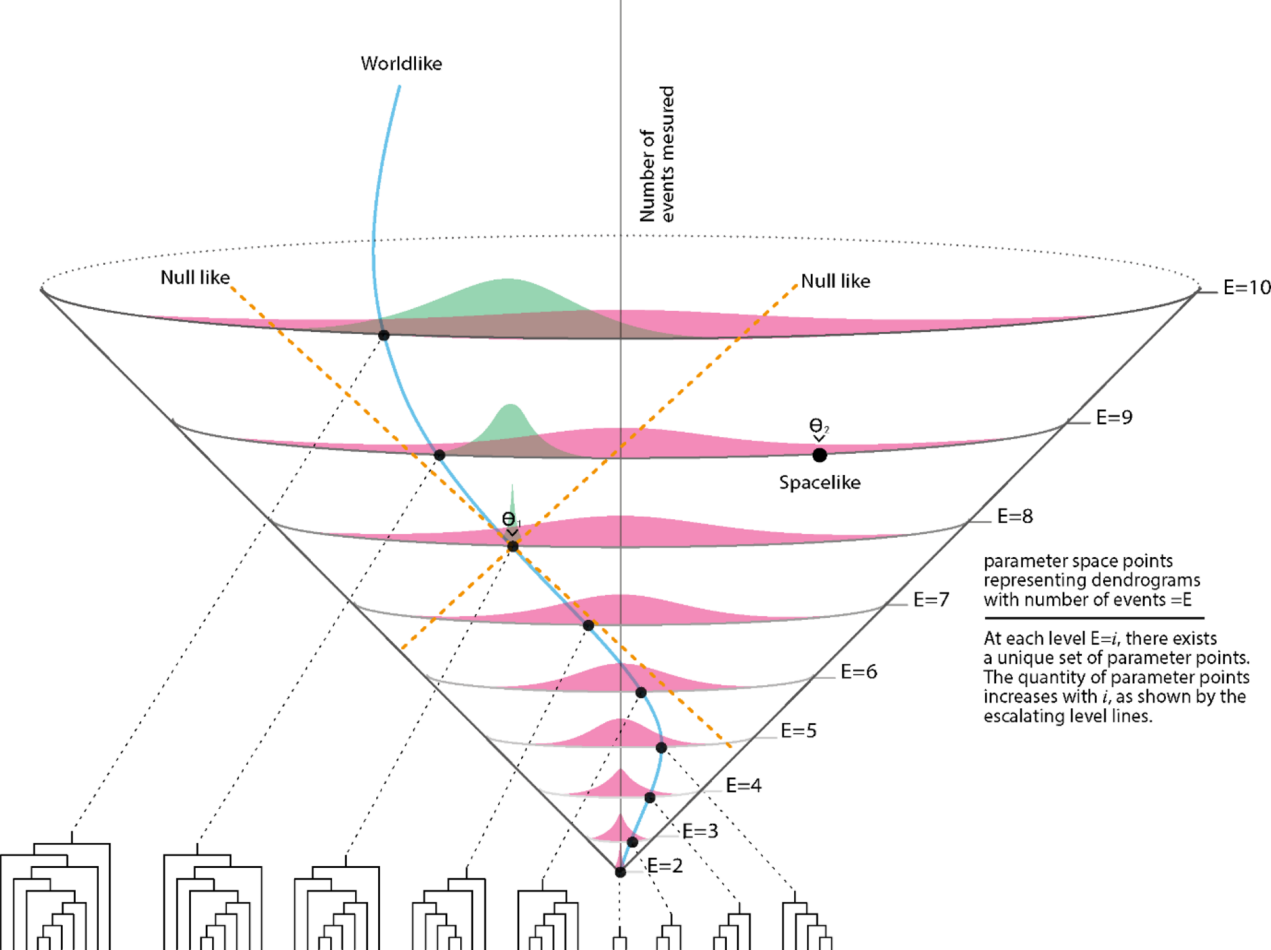
Illustration of the relational information dendrogramic space. Initially, all N observers have a trivial dendrogram with two branches at E = 2. When observers measure more events, they evolve their dendrograms. The possible number of dendrogram structures increases with number of events level E. the distribution of N observers, initially all concentrated with a single structure at E = 2, over the possible dendrogram structure at each level E_i_ is described by the pink distributions. The blue line with black dots depicts the world line of a single observer traversing the parameter space. Each dot on the world line represents a unique dendrogram structure outlined below for each level E_i_ i = 2–10. The point $\theta 1$ is also a point where some fraction of the N observers will pass through. This fraction of observers is described by the value of the pink distribution at $\theta 1$. When $\theta 1$, the fraction of observers passing through it will evolve their dendrograms only within a partial section of the parameter space. The parameter points within this partial section are time-like to that point. Again initially the whole fraction of the observers is concentrated at $\theta 1$. Upon measuring more events, the potential number of dendrogram structures that the fraction of observers will develop increases. Thus, the initial population of observers at $\theta 1$ is described by the green distributions. None of the observers at $\theta 1$ will pass through points outside their light cone, such as $\theta 2$, making $\theta 2$ a space-like point for the observers passing at $\theta 1$.

Initially, all N observers have a trivial dendrogram with two branches. As observers accumulate more events, various dendrogram configurations with more branches become apparent. The number of observers, denoted as $n$, forming a unique dendrogram remains a fraction of the total observers in our universe, $N$, expressed as $\frac{n}{N}$. At each level of $m$ events gathered, all $N$ observers are encompassed. In essence, $n$ is a function of the dendrogram $D$, denoted as $n=n\left(D\right)$. This iterative process enables the evolution of observers’ dendrograms event by event, resulting in increasingly intricate relational structures. Please refer to Fig. 3 for a visual representation. We emphasize that in the current model any observer can measure any event at any iterative step increasing the number of events relationally represented in the dendrogram.

In essence, for two observers dendrograms, $D1\, and\, D2$, which gathered each a set of $m$ events, $set1\, and\, set2,$ there are 3 possibilities

1. $$set1\cap set2=set1 \cup set2$$ leading to $$D1=D2$$.
2. $set1\cap set2=\varnothing$ but the set of relations between events in $set1$ is equal to the set of relations between events in $set2$ leading to $D1=D2$.
3. $set1\cap set2=\varnothing$ and the set of relations between events in $set1$ is not equal to the set of relations between events in $set2$ leading to $D1\ne D2$.
4. $set1\cap set2\ne \varnothing$ but the set of relations between events in $set1$ is equal to the set of relations between events in $set2$ leading to $D1=D2$.
5. $set1\cap set2\ne \varnothing$ and the set of relations between events in $set1$ is not equal to the set of relations between events in $set2$ leading to $D1\ne D2$.

A given dendrogram might have some dendrograms they can evolve to and some they will not. This is the case if a fraction of the observers with dendrogram $D1$ evolves to a dendrogram $D2$ or otherwise none of the observers with $D1$ evolve to $D2$. The question that arises is whether by examining the structure of two dendrograms, can we predict whether they are connected? Dendrograms that exhibit observer flow from one to the other can be referred to as “timelike separated” dendrograms, while dendrograms that show no observer flow between them can be classified as “spacelike separated”.

More specifically, “timelike/spacelike separated” dendrograms are defined as follows:

### Definition 1

$D1$ *and* $D2$ *are two*,* timelike separated*,* different dendrograms with number of events/data collected* $e1\le e2$, *respectively*,* if and only if there exist at least one observer with D1 dendrogram with* $e1$ *events moves from* $D1$ *to* $D2$ *upon collecting the next* $e2-e1$ *events.*

### Definition 2

$D1$ *and* $D2$ *are two*,* spacelike separated*,* different dendrograms with number of events/data collected* $e1\le e2$, *respectively*,* if and only if there are zero observers with* $D1$ *dendrogram with* $e1$ *events to move from* $D1$ *to* $D2$ *upon collecting the next* $e2-e1$ *events.*

*More simply*, consider a scenario *with* $N\to {\infty }$ observers. After measuring $m$ events and representing their relations as a dendrogram, a finite fraction $\frac{n}{N}>0$ (thus $n$ is not finite) of the N observers acquire a unique dendrogram $D1$. Now, consider a unique dendrogram $D2$ representing relations of $m+k$ events ( $k$ =1,2…).

*Timelike separated* means: *at least one observer* out of the $n$ observers, all with $D1$ dendrogram, after measuring another $k$ events acquire the $D2$ dendrogram.

*Spacelike separated* means: *none of the* $n$ *(infinite) observers*, all with $D1$ dendrogram, after measuring another $k$ events acquire the $D2$ dendrogram. We note that all different unique dendrograms at the same level

$\text{m}=\text{m} \,\text{events measured}$ are by definition spacelike separated.

In essence, we will introduce the concept of a dendrogramic “light cone,” first:

### Definition 3

*A specific dendrogram* $D$ *is identified with numerous observers who share identical relations among all the observations each of them has made of the universe.*

Thus, a dendrogramic “light cone” is defined as:

### Definition 4

*A dendrogramic “future light cone” associated with a particular unique dendrogram*, $D$, *encompasses all the potential dendrograms that can evolve from dendrogram* $D$.

*A dendrogramic “past light cone” associated with a particular unique dendrogram*, $D$, *encompasses all past dendrograms that could have evolved to dendrogram* $D$.

More simply, Consider a scenario *with* $N\to {\infty }$ observers. After measuring $m$ events and representing their relations as a dendrogram, a finite fraction $\frac{n}{N}>0$ (thus $n$ is not finite) of the $N$ observers acquire a unique dendrogram $D$.

Those $n$ observers all possible future dendrograms upon acquiring additional any $k=\text{1,2}.\to {\infty }$ events constitutes the future light cone of the unique dendrogram $D.$

all possible past dendrograms of $n$ observers with any $k=\text{2,3}\dots m$ events constitutes the past light cone of the unique dendrogram $D$.

This holds true irrespective of the observer’s identity in relation to $D$ and considers all conceivable events and combinations of events they might measure.This draws an analogy to the Minkowski spacetime metric that characterizes events. This concept allows us to analyze the propagation of relational information of an observer or ensemble of observers within the dendrogramic framework.

Please note that while the causal structure of Minkowski space is not statistical in nature, in DHT, we seek to establish a statistical counterpart referred to as the *“dendrogramic Minkowski causal structure of observers ensemble relational Information universe “*.

Where a *universe* is defined as:

### Definition 5

*A universe is unique dendrogram* $D$. *Thus*, a *universe is an observer’s*,* current*,* relational knowledge (information) he acquired on the ontic universe by measuring some finite amount of events. Moreover*,* by definition* 3*and* 4, *a current unique dendrogram* $D$ *representing relations of* $m$ events is *acquired by* a finite fraction $\frac{n}{N}>0$ ( where - $N\to {\infty }$ and thus $n$ is not finite) *observers. As a consequence*,* all* $n$ *observers have the same universe after measuring* $m$ events.

Thus, for a finite dendrogram the *dendrogramic Minkowski space does not allow us to uniquely define an observer. As a consequence*,* it does not allow us to determine for a single observer which dendrograms will he acquire in each step.*

We emphasize again, each dendrogram represents a fraction of observers who collect a specific number of events (e.g., level) this fraction of observers have same relations between their acquired events. Thus, we operate within statistical ensemble of observers that can measure any possible event from an ensemble of events at any iterative step increasing the number of events relationally represented in the dendrogram. Each unique dendrogram is identified with numerous observers and representing the same relations between the events they measured (generally not same sets of events).

### Real parametrization of dendrograms-lossless compression

In our study, we employed the following equation to facilitate our analysis.

The representation of a dendrogram branch, denoted as ${edge}_{i}$, can be expressed as the sum of a series:

1.1 $${edge}_{i}={\sum }_{j=0}^{k}{a}_{j}\times {p}^{j},{a}_{j}=\text{0,1}.p-1.$$

Throughout this study we will use *p* = 2 thus ${a}_{j}=\text{0,1}$

Here, ${a}_{j}$ represents the binary digit at position j, with possible values of 0 or 1.

we now introduce the concept of the monna map conversion of an edge to event, denoted as ${event}_{i}$, is computed using the formula:

1.2 $${event}_{i}={\sum }_{j=0}^{k}{a}_{j}\times {p}^{-j-1}, {a}_{j}=\text{0,1}.p-1.$$

Throughout this study we will use p=2 thus ${a}_{j}=\text{0,1}$

where ${a}_{j}$ represents the binary digits (0 or 1) in the 2-adic expansion of the dendrogram branch, and k is the maximum ball level of the dendrogram.

By applying this Monna map conversion, we represent the edges as rational numbers on the continuous interval [0,1]. This conversion preserves the precise relations between the edges, ensuring that the inherent structure and ordering within the dendrogram branches are maintained. Furthermore, we introduced the metric ${q}_{ik}$, which represents the absolute difference two events,

1.3 $${q}_{ik}=\left|{event}_{i}-{event}_{k}\right|$$

we can define 5 parameters of a dendrogram as follows.

First we define our dendrogramic vector, $D$, as follows:

$$E={event}_{i },\ where\  i=\text{1,2}\dots n=number\,of \,events$$

$$B={2}^{-maximal\, ball\, level\, of\, the\, dendrogram}$$

$$D=\left[E\  B\right]\, with\, elements\, {D}_{i },\ i=\text{2,3}\dots n+1$$

$${V}_{D}=({\sum }_{i=0}^{k}{D}_{i }{)}^{z}$$

$${U}_{D}=({\sum }_{i=0}^{k}\frac{1}{{D}_{i }+1}{)}^{z1}$$

$${M}_{D}=(\sum _{i=1}^{k-1}\sum _{j=i+1}^{k}{D}_{i } \cdot {D}_{j }{)}^{z2}$$

$${R}_{D}=(\sum _{i=1}^{k-1}\sum _{j=i+1}^{k}{D}_{i }- {D}_{j }{)}^{z3}=(\sum _{i=1}^{k-1}\sum _{j=i+1}^{k} {q}_{ij }{)+\sum _{j=i+1}^{k}\left|B- {D}_{j}\right|)}^{z3}$$

1.4 $${r}_{D}=(\sum _{i=1}^{k-1}\sum _{j=i+1}^{k}{1/\left(\right(D}_{i }- {D}_{j })+1){)}^{z4}=(\sum _{i=1}^{k-1}\sum _{j=i+1}^{k} {1/(q}_{ij }+1){+\sum _{j=i+1}^{k}1/(\left|B- {D}_{j}\right|+1\left)\right)}^{z4}$$

$$k=number\, of\, branches \,and\, thus\, events\, in\, dendrogram \,and$$

$$where\, z,z1,z2,z3 \,and \,z4 \,\text{are}\, \text{f}\text{r}\text{e}\text{e}\, \text{p}\text{a}\text{r}\text{a}\text{m}\text{e}\text{t}\text{e}\text{r}\text{s}$$

we will demonstrate now that there are parameters spaces that have the ability to uniquely define a unique dendrogram structure. For:

$${{\theta }^{{\prime }}}_{1}={(U}_{D}{)}^{-2}$$

$${{\theta }^{{\prime }}}_{2}={(V}_{D}{)}^{0.5}/{(U}_{D}{)}^{-2}$$

$${{\theta }^{{\prime }}}_{3}={(U}_{D}{)}^{-2}{{(V}_{D}{)}^{0.5}{{R}_{D}}^{-0.5}(M}_{D}{)}^{2}$$

$${ \theta^{\prime }}_{4}=\sqrt{n}({r}_{D}{)}^{2}/{{R}_{D}}^{-0.5}$$

*Lets suppose* ${\theta ^{\prime }}_{1}={\theta ^{\prime \prime }}_{1} , {\theta ^{\prime }}_{2}={\theta ^{\prime \prime }}_{2}, {\theta ^{\prime }}_{3}={\theta ^{\prime \prime }}_{3} \,and\, {\theta ^{\prime }}_{4}={\theta ^{\prime \prime }}_{4}$ *for two different dendrograms* $D^{\prime } \,and\, D^{\prime \prime }$ *then if* ${\theta ^{\prime }}_{1}={\theta ^{\prime \prime }}_{1} \,and\,$ ${\theta ^{\prime }}_{2}={\theta ^{\prime \prime }}_{2}$ *then* ${(U}_{D^{\prime }}{)}^{-2}={(U}_{D^{\prime \prime }}{)}^{-2}$ *and*.

$\frac{{(V}_{{D}^{{\prime }}}{)}^{0.5}}{{(U}_{{D}^{{\prime }}}{)}^{-2}}=\frac{{(V}_{{D}^{{\prime \prime }}}{)}^{0.5}}{{(U}_{{D}^{{\prime \prime }}}{)}^{-2}}$ *which means* ${(V}_{{D}^{{\prime }}}{)}^{0.5}={(V}_{{D}^{{\prime \prime }}}{)}^{0.5}$ *the combination*.

$$\left\{\begin{array}{c}{(V}_{{D}^{{\prime }}}{)}^{0.5}={(V}_{{D}^{{\prime \prime }}}{)}^{0.5}\\ {(U}_{D^{\prime }}{)}^{-2}={(U}_{D^{\prime \prime }}{)}^{-2}\end{array}\right.$$ *can’t happen by their definition unless* ${D}^{{\prime }}= D^{\prime \prime }$ *and we are done.*

We’ve demonstrated that relational information, represented by strings or p-adic expansions, can be compressed without loss into a 4-dimensional parameter point.

### The informational Minkowski-like metric of the relational information dendrogramic space

Having established the existence of at least one space with four parameters that uniquely determine a dendrogram, we can now develop an informational metric inspired by Minkowski spacetime. We will describe our model step by step: each parameter point $\theta$ in parameter space uniquely defines a dendrogram with ${n}_{\theta }$ events. This dendrogram is the n level state of an ensemble of observers with same dendrogram. Thus, the point $\theta$ has a flow-in of observers from different smaller dendrograms with $n-1$ events. The distribution of observers over these smaller dendrograms can be converted to an observer’s distribution over ${\varvec{\theta }}^{\varvec{{\prime }}}.$ where ${\varvec{\theta }}^{\varvec{{\prime }}}$ are parameter points of all dendrograms with $n-1$ events. This is the past observer distribution of $\theta$

Furthermore, the dendrogram at level n, at point $\theta$, exhibits a flow of observers emanating from the $\theta$ parameter point. The ensemble of observers, at $\theta$, is distributed to the next level dendrograms with $n+1$ events. This certain manner of distribution of the observers represents the future distribution of the n-level $\theta$ point.

Specifically, for a single dendrogram at level n (representing a fraction of $m/N$ observers), its future cone at levels $n+k$ (where $k$ ranges from 1 to $M\to {\infty }$ ) consists of k distributions ${\rho }_{k,n}({\theta }^{{\prime }}{)}_{future}$, dependent on the level $n$ (or number of events in dendrogram) of the initial ${\theta }^{{\prime }}$ point in the parameter. Similarly, the past cone of a dendrogram at level $n$ extends to level $n-k$ (where $k$ ranges from 1 to $n-2$ ) and exhibits $k$ distributions ${\rho }_{k,n}({\theta }^{{\prime }}{)}_{past}$ (see Fig. 3).

#### Ontic–epistemic linkage

consider a finite fraction $\frac{m}{N}\, (where \,N\to {\infty })$ of observers with same unique dendrogram $D$, with finite $n$ events ( ${n}^{th}$ level), represented by a point ${\theta }^{{\prime }}$ in parameter space. $D$ is finite and thus Epistemic dendrogram, relational structure. When each of those $m$ observers acquire another $k\to {\infty }$ events the distributions ${\rho }_{k,n}({\theta }^{{\prime }}{)}_{future}$ at $n+k$ flatten, implying each observer possesses a unique infinite dendrogram. Thus for $k\to {\infty }$, point ${\theta }^{{\prime }}$ distribution ${\rho }_{k,n}({\theta }^{{\prime }}{)}_{future}=f\left({{\varvec{\theta }}^{\varvec{{\prime }}}}_{\mathbf{\infty }}\right)=\frac{1}{m}.$

In fact the epistemic point ${\theta }^{{\prime }}$ is defined by a $m$ size set of infinite ontic dendrograms and vice versa.

Consider two epistemic points ${\theta }^{{\prime }}1$ and ${\theta }^{{\prime }}2$ with $m1\ and\ m2$ number of observers and thus with $m1\ and\ m2$ size set of infinite ontic dendrograms. An intersection between the ${\theta }^{{\prime }}1$ and ${\theta }^{{\prime }}2$ sets means observers transitioning between those points-thus ${\theta }^{{\prime }}1$ and ${\theta }^{{\prime }}2$ are timelike separated. Conversely, an empty intersection signifies spacelike separation between ${ \theta }^{{\prime }}1$ and ${\theta }^{{\prime }}2$.

If we consider observers and worldline as entities then, as shown above, the ontic Leibnitz principle is followed. Moreover, those arguments imply:

1. Distinguishability by ontic Leibnitz principle $\leftrightarrow$ 2. observer’s ontic dendrogram uniqueness $\leftrightarrow$ 3. uniqueness of observer infinite worldline through parameter space.

We note that an infinite worldline is equivalent to an observer infinite dendrogram which, in turn, is a unique infinite but partial set of allowed relational branches out of the whole p-adic tree.

We need to stress again the linkage- an epistemic dendrogram relational structure at ${\theta }^{{\prime }}$ is fully defined by a set of ontic infinite dendrograms and thus a set of infinite worldlines.

Please note that an infinite as well as finite dendrograms are a relational structures and thus, in the current model presented, views from epistemic relationism, ontic relationism as well as Platonist views reside together without much contradictions.

We stress that a point $\theta$ in parameter space is accompanied with all its past distributions flowing into that point and all future distribution flowing out of that point. These distributions and the point $\theta$ are defined either by the set of the observers that flow in and out of the $\theta$ point or the set of ultimate infinite dendrograms, defining unique observers, and vice versa.

We can envision the distributions dynamics as manifested from level to level by a “potential”/”force” we call the “Leibnitz potential/force” which forces the observers to become distinct (ultimately at the ontic infinite sub dendrogram), Otherwise the ontic Leibnitz principle holds. Now we can modify the epistemic principle into an ontic dendrogramic reformulation: *If*,* for every dendrogram D∞*,* observer x has D∞ if and only if observer y has D∞*,* then observer x is identical to observer y.*

To quantify the informational distance between two parameter points accompanied with their distribution, we propose an informational geometric metric:

1.5 $$informational \,distance=2{H}_{f}^{\left\lceil T \right\rceil}+2{H}_{p}^{\left\lceil T \right\rceil}-\left({H}_{{f}^{{\prime }}}+{H}_{{p}^{{\prime }}}\right)-2\left(\frac{L}{L+1}{H}_{ratio}+\left\lceil T \right\rceil\right)$$

**Fig. 4:**
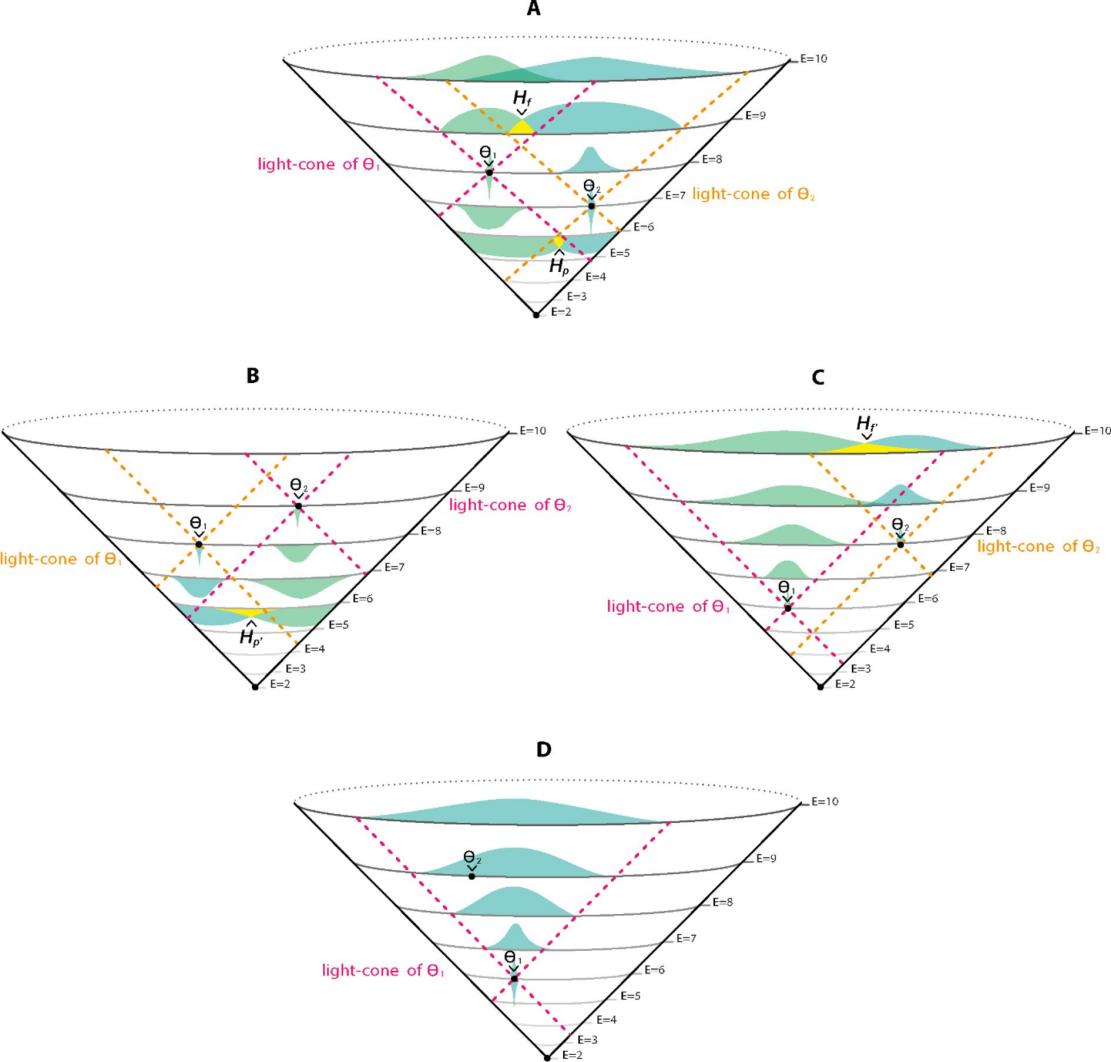
Illustration of components in the informational geometric metric $Informational\, distance=2{H}_{f}^{\left\lceil T \right\rceil}+2{H}_{p}^{\left\lceil T \right\rceil}-\left({H}_{{f}^{{\prime }}}+{H}_{{p}^{{\prime }}}\right)-2\left(\frac{L}{L+1}{H}_{ratio}+\left\lceil T \right\rceil\right) .$ (**A**) Illustration of two informational light cones originating at $\theta 1 \,and \,\theta 2$. ${H}_{f} \,and\, {H}_{p}$ are calculated from the yellow intersections (indicated by ${H}_{f}\, and\, {H}_{p}$ and arrow) of the green and blue distributions belonging to $\theta 1\, and\, \theta 2$ respectively. (**B**) Illustration of two informational light cones originating at $\theta 1\, and\, \theta 2$. ${H}_{p^{\prime }}$ is calculated from the yellow intersection (indicated by ${H}_{p^{\prime }}$ and arrow) of the blue and green distributions belonging to $\theta 1 \,and\, \theta 2$ respectively. (**C**) illustation of two informational light cones originating at $\theta 1\, and\, \theta 2$. ${H}_{f^{\prime }}$ is calculated from the yellow intersection (indicated by ${H}_{f^{\prime }}$ and arrow) of the blue and green distributions belonging to $\theta 1\, and\, \theta 2$ respectively. (**D**) illstration of the $T$ component calculated for $\theta 1\, and \,\theta 2$. value (indicated by an arrow) of the blue distribution value at $\theta 2.$

Consider two points ${\theta }^{{\prime }}1\, \text{a}\text{n}\text{d}\, {\theta }^{{\prime }}2 \,\text{w}\text{h}\text{e}\text{r}\text{e}\, n1, n2 \,\text{a}\text{r}\text{e}\, \text{t}\text{h}\text{e}\text{i}\text{r}\, \text{l}\text{e}\text{v}\text{e}\text{l}$ where without loss of generality $n1\le n2$ :

${H}_{f}=Hellinger\, distance\, between$ ${\rho }_{k,n1,{\theta }^{{\prime }}1}(x{)}_{future at n2+1}\, and$ ${\rho }_{k,n2,{\theta }^{{\prime }}2}(x{)}_{future\, at\, n2+1}$ (Fig. 4A), *if* ${\rho }_{k,n1,{\theta }^{{\prime }}1}(x{)}_{future\, at\, n2+1} \,and$ ${\rho }_{k,n2,{\theta }^{{\prime }}2}(x{)}_{future \,at\, n2+1}$ *share parameter points with non zero probability* ${0\le H}_{f}<1$ *else* ${H}_{f}=1$

${H}_{p}=$ ${Hellinger\, distance\, between\, \rho }_{k,n1,{\theta }^{{\prime }}1}(x{)}_{past \,at\, n1-1}\, and$ ${\rho }_{k,n2,{\theta }^{{\prime }}2}(x{)}_{past \,at \,n1-1}$ (Fig. 4A), *if* ${\rho }_{k,n1,{\theta }^{{\prime }}1}\,(x)_{past\, at\, n1-1}\, and\,{ \rho }_{k,n2,{\theta }^{{\prime }}2}\,(x{)}_{past \,at\, n1-1}$ *share parameter points with non zero probability* ${0\le H}_{f}<1$ *else* ${H}_{f}=1$

$$\dddot{{\rho }_{k,n1,{\theta }^{{\prime }}1}}(x{)}_{ future\, n1+k1 }=the \,minimum\, k1\, value\, distribution \,of \,{\theta }^{{\prime }}1$$

$${\rho }_{k,n2,{\theta }^{{\prime }}2}(x{)}_{future \,n2+k2}=the \,minimum\, k2\, value \,distribution\, of \,{\theta }^{{\prime }}2$$

$$where \,both \,distributions\, intersect\, with\, nonzero\, probability\, values$$

*Thus*: ${H}_{f{\prime }}=$ *Hellinger distance between* $\dddot{{\rho }_{k,n1,{\theta }^{{\prime }}1}}(x{)}_{ future\, n1+k1 }$ *and* ${\rho }_{k,n2,{\theta }^{{\prime }}2}(x{)}_{future\, n2+k2}$ (Fig. 4C)

$$\dddot{{\rho }_{k,n2,{\theta }^{{\prime }}2}}(x{)}_{ past \,n2-k2 }= the \,minimum \,k2\, value\, distribution\, of\, {\theta }^{{\prime }}2$$

$${\rho }_{k,n1,{\theta }^{{\prime }}1}(x{)}_{past\, n1-k1}=the\, minimum\, k1\, value\, distribution \,of\, {\theta }^{{\prime }}1$$

$$where\, both\, distributions \,intersect\, with\, nonzero\, probability\, values.$$

*Thus*: ${H}_{p{\prime }}=$ *Hellinger distance between* $\dddot{{\rho }_{k,n2,{\theta }^{{\prime }}2}}(x{)}_{ past\, n2-k2}$ *and* ${\rho }_{k,n1,{\theta }^{{\prime }}1}(x{)}_{past\, n1-k1}$ (Fig. 4B)

$$\text{we define:}\  L=|n1-n2|$$

$${H}_{ratio}=Hellinger\, distance\, of\, two\, distributions\, {p}_{pop {\theta }^{{\prime }}1} \,and\, {p}_{pop {\theta }^{{\prime }}2}$$

*Where* $${p}_{pop {\theta }^{{\prime }}1}=\left[\frac{number \,of\, observers\, at\, point\, {\theta }^{{\prime }}1 \,at\, level\, n1 }{total \,number \,of\, observers} \frac{number \,of \,observers\, at\, points \,other \,then\, {\theta }^{{\prime }}1 \,at\, level\, n1 }{total \,number \,of \,observers}\right]$$

$${p}_{pop {\theta }^{{\prime }}2}=\left[\frac{number \,of \,observers\, at\, point\, {\theta }^{{\prime }}2\, at\, level\, n2 }{total\, number\, of\, observers} \frac{number\, of\, observers\, at\, points\, other\, then\, {\theta }^{{\prime }}2 \,at\, level\, n2 }{total \,number \,of\, observers}\right]$$

$$\text{finally}\, \text{we}\, \text{define:}\, T={p}_{{\theta }^{{\prime }}1}(x={\theta }^{{\prime }}2)=distribution\, value\, of\, {\rho }_{k,n1,{\theta }^{{\prime }}1}(x{)}_{future \,at\, n2}\, at \,x={\theta }^{{\prime }}2$$ (Fig. 4D).

Now for proving the timelike/spacelike signature:

For time like dendrograms ${H}_{f}$ = ${H}_{{f}^{{\prime }}}$, ${H}_{p}={H}_{{p}^{{\prime }}}$ and $\left\lceil T \right\rceil=1$. Thus, the metric reduces to: $informational\, distance={H}_{f}+{H}_{p}-2\left(\frac{L}{L+1}{H}_{ratio}+\left\lceil T \right\rceil\right).$

${0\le H}_{f}+{H}_{p}<2$ since they are timelike. On the other hand: ${H}_{ratio}\le 1 , \frac{L}{L+1}<1$

As $\left\lceil {p}_{{\theta }^{{\prime }}1}\left(x={\theta }^{{\prime }}2\right)\right\rceil=\left\lceil T \right\rceil=1$ resulting from the fact that time like dendrograms share some fraction of observer flow from one dendrogram to the other.

So, the component $2\le 2\left(\frac{L}{L+1}{H}_{ratio}+\left\lceil T \right\rceil\right)$ resulting in:

$${H}_{f}+{H}_{p}-2\left(\frac{L}{L+1}{H}_{ratio}+\left\lceil T \right\rceil\right)<0$$

Now for space like, we have in fact two cases. lets treat the first one where n1 < n2.

${H}_{f}=1, {H}_{p}=1$ thus, we reduce the metric to ${4-(H}_{f{\prime }}+{H}_{p{\prime }})-2\left(\frac{L}{L+1}{H}_{ratio}+\left\lceil T \right\rceil\right).$

Since: $${0<(H}_{f{\prime }}+{H}_{p{\prime }})\le 2-\to {4-(H}_{f{\prime }}+{H}_{p{\prime }})\ge 2$$

Then for $2\left(\frac{L}{L+1}{H}_{ratio}+\left\lceil T \right\rceil\right)$ we have ${H}_{ratio}\le 1 , \frac{L}{L+1}<1$ but $\left\lceil {p}_{{\theta }^{{\prime }}1}\left(x={\theta }^{{\prime }}2\right)\right\rceil=\left\lceil T \right\rceil=0$ so $2\left(\frac{L}{L+1}{H}_{ratio}+\left\lceil T \right\rceil\right)<2$ thus, the interval is greater than zero.

The other case is when ${H}_{f}<1, {H}_{p}<1$ then ${H}_{f}$ = ${H}_{{f}^{{\prime }}}$, ${H}_{p}={H}_{{p}^{{\prime }}}$ and $\left\lceil T \right\rceil=0$ leading to

$$informational\, \,distance={4-(H}_{f}+{H}_{p})-2\left(\frac{L}{L+1}{H}_{ratio}+\left\lceil T \right\rceil\right)$$

Again ${0\le H}_{f}+{H}_{p}<2$ thus ${4-(H}_{f{\prime }}+{H}_{p{\prime }})\ge 2$ but $2\left(\frac{L}{L+1}{H}_{ratio}+\left\lceil T \right\rceil\right)$ reduces to

$$2\left(\frac{L}{L+1}{H}_{ratio}\right)<2$$

Thus: ${4-(H}_{f^{\prime }}+{H}_{p^{\prime }})-2\left(\frac{L}{L+1}{H}_{ratio}+\left\lceil T \right\rceil\right)>0$ and again, the interval is bigger then zero

For n1 = n2.

We have if ${H}_{f}=1, {H}_{p}=1-\to 4$ ${-(H}_{f^{\prime }}+{H}_{p^{\prime }})-2\left(\frac{L}{L+1}{H}_{ratio}+\left\lceil T \right\rceil\right)$ since $\left\lceil T \right\rceil=0$ and $L=0$ thus $2\left(\frac{L}{L+1}{H}_{ratio}+\left\lceil T \right\rceil\right)$ =0 and we have $4$ ${-(H}_{f^{\prime }}+{H}_{p^{\prime }})\ge 2$ and we are done.

If we have ${H}_{f}<1, {H}_{p}<1-\to 4$ ${-(H}_{f^{\prime }}+{H}_{p^{\prime }})-2\left(\frac{L}{L+1}{H}_{ratio}+\left\lceil T \right\rceil\right)$ since $\left\lceil T \right\rceil=0$ and $L=0$ where ${H}_{f}$ = ${H}_{{f}^{{\prime }}}$, ${H}_{p}={H}_{{p}^{{\prime }}}$ we conclude that $2\left(\frac{L}{L+1}{H}_{ratio}+\left\lceil T \right\rceil\right)$ =0 and we have ${4-(H}_{f^{\prime }}+{H}_{p^{\prime }})\ge 2$

and we are done.

### Establishing dendrogram-parameters coupling via numerical simulation

Although the informational geometric Minkowski-like metric is analytically proven in practical data analysis, distinguishing “time-like” and “space-like” dendrograms remains a challenge. To address this, we need parameter spaces reflecting Minkowski space-time, exhibiting light-cone characteristics. proving analytically that a real parameterization encoding of a dendrogram poses a Minkowski-like character, is complex and requires significant time. In the realm of DHT theory, we propose “numerical experimenting confirmation” to select parameters, validated through extensive simulations. While not a mathematical proof, the likelihood of encountering dendrograms that do not conform to our parametrization is practically negligible. We outline our procedural methods used in numerical simulations, along with the results and conclusions, in the appendix sections A1.1-A1.3.

The consequence of our numerical analysis. We propose that:

1.6 $${p}_{{\theta }^{\varvec{{\prime }}}}\left(X\right)=\frac{1}{(2\pi {a}^{2}{)}^{2}}\text{exp}\left(-\frac{(t-i{{\left(s2\right)\theta }^{{\prime }}}_{4}{)}^{2}+(x-{{\theta }^{{\prime }}}_{1}{)}^{2}+(y-{{\theta }^{{\prime }}}_{2}{)}^{2}+(z-{{\theta }^{{\prime }}}_{3}{)}^{2}}{2{a}^{2}}\right)$$

*Where* $X$ *is a vector of* $[t,x,y,z]$ *and* $s2$ *is our equivalent of* $c=speed\, of\, light$.

### This distribution is normalized: meaning $\int {d}^{4}X{p}_{{\theta }^{{\prime }}}\left(X\right)=1$*and leads to* a fisher information matrix

1.7 $${g}_{\mu \nu }=\left(\begin{array}{cccc}-s2& 0& 0& 0\\ 0& 1& 0& 0\\ 0& 0& 1& 0\\ 0& 0& 0& 1\end{array}\right)$$

After rescaling by ${a}^{2}$ ^38^.

Furthermore, we define *n*_*i*_ $=number \,of \,events\, in\, dendrogram\, i, i^{\prime}=\text{2,3}\dots$ and $N=|n1-n2|$ then we define the simultaneity matrix or operator of these Minkowski-like parameter spaces

1.8 $$\widehat{sy{m}_{+}}= \left(\begin{array}{cccc}\lceil \frac{N}{N+1}\rceil & 0& 0& 0\\ 0& 1& 0& 0\\ 0& 0& 1& 0\\ 0& 0& 0& 1\end{array}\right)$$

And thus:

1.9 $$dist\left(dendrogram1,dendrogram2\right)=(\widehat{sy{m}_{+}}{{)}_{\mu \nu }g}_{\mu \nu }d{{\theta }^{{\prime }}}_{\mu }d{{\theta }^{{\prime }}}_{\nu }$$

One benefit of this construction is the transformation of the discrete parameter space into a continuous one. Thus, every dendrogram is defined by its non-hermitian distribution ${p}_{{\theta }^{\varvec{{\prime }}}}\left(X\right)$ and we operate on a smooth Riemann 4d parameter space. This will result also in null like parameters for a given dendrogram (although they will (probably) not represent a dendrogram relational structure)

### Emergent many worlds interpretation in the relational information framework

In this section, we view events as interactions or measurements of an observer upon another. We’ve established, in the previous section, the Minkowski-like parameter space of dendrograms. Thus, a unique observer’s is defined by its unique world line on the parameter space, whether experiencing acceleration or not. As shown in the previous section, two observers cannot share the same infinite world line if adhering to the ontic Leibniz’s principles and vice versa. The observer’s ontic view of the universe relies on the measurements they perform, defining their world line within the subjective parameter space.

Following the epistemic Leibniz’s principle an observer conducts a unique and infinite set of measurements, leading to a distinct world line within the parameter space. Thus, even if the number of observers approaches infinity, each is uniquely defined (by the set of measurements). Defining each observer is achieved by posing an unlimited series of yes/no questions on all world-lines/measurement set and creating an infinite p-adic tree, where each branch represents the relation of an observer world line to all other observer’s world line. This branch is defined as the observer’s objective ontic property.

Thus, events are epistemically measured by one observer. These events are other observer’s ontic objective properties. We stress that subjective dynamic of an observer is guided by the objective properties of other observers he measures. In turn the objective properties of each of these observers are their infinite worldline- infinite subjective dynamic. An infinite worldline is equivalent to an observer infinite dendrogram which, in turn, is a unique infinite but partial set of allowed relational branches set out of the whole p-adic tree ( see section Ontic-Epistemic linkage above).

Thus: unique infinite partial set of allowed relational branches set out of the whole p-adic tree is an objective property of an observer.

Again, a connection between the ontic (the p-adic infinite tree) and the observer’s subjective- epistemic view is established.

Moreover, in this model, the observer/system discrepancy converges into an all-encompassing observer universe, where every physical entity interacting or rather measuring, with another is considered an observer. We will show also that the “observer” and “observed” (measured) apply to any arbitrary system, microscopic or macroscopic.

### The observer subjective wave function

The construction of the observer subjective wave function was developed thoroughly in recent work^5^.

We emphasize that the subjective wave function, ${\psi }_{subjective}$, is completely dependent on the measurements the observer is preforming thus for a set *M= {m*_*1*_,*m*_*2*_. *m*_*i*_*}*,

${\psi }_{subjective}\left(M\right)={\psi }_{subjective}\left(\varvec{\theta }\right)$ where different sets M can have same $\varvec{\theta }$.

The transition of this kind of measurements “world line” into the dynamical evolvement of the subjective wave function, ${\psi }_{subjective}\left(\varvec{\theta }\right)$, is shown in^5^ and is emergent from the following action through the bohmian mechanics formalism:

2.1 $$A\left(S,\rho \right)=\int d\left(\varvec{\theta }\right)\{\int \frac{d\left(S\right)}{d\varvec{\theta }}\rho \left(Q\right)dQ+\int {(\partial S)}^{2}\rho \left(Q\right)dQ-{\upnu }\left(Q\right)+U\left(Q\right)\}$$

For a detailed derivation of Eq. 2.1 we refer the reader to^5^ or the Supplementary Appendix Section A2.1.

### Transformation of objective property of an observer to wave function

Let us consider the situation where all observers at a particular $\varvec{\theta }$ measure a particular observer ${O}_{{B}_{k}}$. The objective property of ${O}_{{B}_{k}}$ is composed as follows : the sum of the finite p-adic expansion with x_0_ , x_1_ x_2_  ....x_k,_ is ${Z}_{{B}_{k}}$ meaning the objective property is a p-adic ball. This p-adic expansion can be transformed by monna map to a rational number ${q}_{{B}_{k}}\in \left[0\ 1\right]\subset \mathbb{Q}$.

Each of the observers at $\varvec{\theta }$ measure the same ${q}_{{B}_{k}}$ and incorporate it subjectively into their dendrogram. Thus, each observer at $\varvec{\theta }$ will incorporate it as a finite branch with different p-adic expansion that by the monna map will be identified with a rational number value, we call event, on the interval $\left[0\ 1\right]$. So, for $\varvec{\theta }$ ${O}_{{B}_{k}}$ is a distribution of possible rational numbers. we thus identify ${O}_{{B}_{k}}$ as a distribution ${\rho }_{{B}_{k}}\left(x,\varvec{\theta }\right)$ on the interval $x\in \left[0\ 1\right]$. From these possible events values we can construct the objective wave function of ${O}_{{B}_{k}}$ for $\varvec{\theta }$ **.** At some level $n,$ the procedure to construct the objective wave function of ${O}_{{B}_{k}}$ follows same procedures as in^5^ and section A2.1 (Eq. 2.1–2.10). In this procedure, ${event}_{i}={\sum }_{j=0}^{\infty }{a}_{j}\times {2}^{-j-1}$, ${a}_{j}=\text{1,0}$ is the act of measuring observer ${O}_{{B}_{k}}{\prime }s$ objective property (represented by the ball value that signifies ${O}_{{B}_{k}}$ ) conducted by another observer at $\varvec{\theta }$.

 So, ${\psi }^{{O}_{{B}_{k}}}(\varvec{\theta })=\sqrt{{\rho }_{{B}_{k}}\left(x,\varvec{\theta }\right)}{e}^{iS\left(\varvec{\theta }\right)}$ is a relational wave function (in relation to a certain $\varvec{\theta }$ ). For each different $\varvec{\theta }$ it dynamically changes. this wave function of ${O}_{{B}_{k}}$, is the transformation of an objective property to an ensemble of subjective properties (dependent on the ensemble of observers measuring it) in relation to $\varvec{\theta }.$ Where $\varvec{\theta }$ is inherently subjective, e.g., it represents each of the observers at $\varvec{\theta }$ with same subjective knowledge about the universe.

### Measurement

An observer at $\varvec{\theta }$ has a subjective dendrogram that again (as in our previous study and Sect. 2.1) we can construct from it a subjective wave function ${\psi }^{\varvec{\theta }}$ for the $\varvec{\theta }$ coordinate. For a fraction ${b}_{j}$ of observers at $\varvec{\theta }$, all with same dendrogram, that will measure ${O}_{{B}_{k}}$ and will transform upon this measurement to ${\varvec{\theta }}_{1}$ will have before measurement

2.2 $${\psi }^{{{\varvec{\theta }}^{{b}_{j}}+O}_{{B}_{k}}}=\sum _{i}{{a}_{i}\varphi }_{i}^{{O}_{{B}_{k}}}{\psi }^{\varvec{\theta }}$$

And after

2.3 $${\psi }^{{{\varvec{\theta }}_{1}+O}_{{B}_{k}}}=\sum _{i}{{a}_{i}\varphi }_{i}^{{O}_{{B}_{k}}}{\psi }^{{\varvec{\theta }}_{1}}$$

So, the state of $\varvec{\theta }\left(\varvec{M}\right)$ (level M is the number of edges of the dendrogram that encodes the coordinate $\theta )$ at $\varvec{\theta }(\varvec{M}+1)$ upon all observers, ${b}_{j}$, in $\varvec{\theta }$ measuring ${O}_{{B}_{k}}$ is:

2.4 $${\psi }^{{\varvec{\theta }(\varvec{M}+1)+O}_{{B}_{k}}}=\sum _{j}\sum _{i}{{a}_{i}\varphi }_{i}^{{O}_{{B}_{k}}}{b}_{j}{\psi }^{{\varvec{\theta }}_{\varvec{j}}}$$

Where ${\theta }_{j}\ne \theta$ and $j$ runs from 1 to u

We can now generalize the situation into $\varvec{\theta }\left(\varvec{M}\right)$ measuring several ${O}_{{B}_{k}}{\prime }s$ so $k=\{\text{1,2}.h\}$

The combined distribution of ${O}_{{B}_{\text{1,2}.h}}$ with respect to $\varvec{\theta }$ **is** ${\rho }_{{B}_{\text{1,2}.h}}\left(x,\varvec{\theta }\right)$ and

${\psi }^{{O}_{{B}_{\text{1,2}.h}}}(\varvec{\theta })=\sqrt{{\rho }_{{B}_{\text{1,2}.h}}\left(x,\varvec{\theta }\right)}{e}^{iS\left(\varvec{\theta }\right)}$ with eigenvalues ${\varphi }_{i}^{{O}_{{B}_{\text{1,2}.h}}}$ inserting it to the equation above we have: ${\psi }^{\varvec{\theta }\left(\varvec{M}+1\right)+{O}_{{B}_{\text{1,2}.h}}}=\sum _{j}\sum _{i}{{a}_{i}\varphi }_{i}^{{O}_{{B}_{\text{1,2}.h}}}{b}_{j}{\psi }^{{\varvec{\theta }}_{\varvec{j}}}$ (2.5)

We can even generalize to a region of the parameter space.

So let $$\overline{\varvec{\theta }}\left(\varvec{G}\right)= {\{\varvec{\theta }}_{1}\left(\varvec{k}\right){, \varvec{\theta }}_{2}\left(\varvec{l}\right), { \varvec{\theta }}_{3}\left(\varvec{f}\right)\dots .\}, \varvec{G}=\varvec{k},\varvec{l},\varvec{f}\dots$$

*And the* combined distribution of ${O}_{{B}_{\text{1,2}.h}}$ with respect to $\overline{\varvec{\theta }}$ is ${\rho }_{{B}_{\text{1,2}.h}}\left(x,\overline{\varvec{\theta }}\right)$ and

$${\psi }^{{O}_{{B}_{\text{1,2}.h}}}\left(\overline{\varvec{\theta }}\right)=\sqrt{{\rho }_{{B}_{\text{1,2}.h}}\left(x,\overline{\varvec{\theta }}\right)}{e}^{iS\left(\overline{\varvec{\theta }}\right)} \text{with}\, \text{eigen}\,\text{v}\text{a}\text{l}\text{u}\text{e}\text{s}\, {\varphi }_{i}^{{O}_{{B}_{\text{1,2}.h}}\left(\overline{\varvec{\theta }}\right)}$$

Then we have:

2.6 $${\psi }^{\overline{\varvec{\theta }}(G+1)+{O}_{{B}_{\text{1,2}.h}}\left(\overline{\varvec{\theta }}\right)}=\sum _{j}\sum _{i}{{a}_{i}\varphi }_{i}^{{O}_{{B}_{\text{1,2}.h}}}{b}_{j}{\psi }^{{\overline{\varvec{\theta }}}_{\varvec{j}}}$$

We then can have by Everett second rule another measurement of different set of observers

$${O}_{{B}_{L}} \,\text{w}\text{h}\text{e}\text{r}\text{e} \,L\ne \{\text{1,2}\dots h\} \,\text{r}\text{e}\text{s}\text{u}\text{l}\text{t}\text{i}\text{n}\text{g} \,\text{i}\text{n}$$

2.7 $${\psi }^{\overline{\varvec{\theta }}(G+2)+{O}_{{B}_{\text{1,2}.h}}\left(\overline{\varvec{\theta }}\right)}=\sum _{j}\sum _{l}\sum _{i}{{{{c}_{l}\phi }_{l}^{{O}_{L}}a}_{i}\varphi }_{i}^{{O}_{{B}_{\text{1,2}.h}}}{b}_{j}{\psi }^{{\overline{\varvec{\theta }}}_{\varvec{j}}}$$

We then need to consider the situation where some observer at $\overline{\varvec{\theta }}$ will measure an observer ${O}_{{B}_{m}}$ that he already measured, so his dendrogram will not change and so does his wave function.

Thus

2.8 $${\psi }^{\overline{\varvec{\theta }}\left(G+1\right)+{O}_{{B}_{\text{1,2}.h}}\left(\overline{\varvec{\theta }}\right)+\overline{\varvec{\theta }}}=\sum _{j}\sum _{i}{{a}_{i}\varphi }_{i}^{{O}_{{B}_{\text{1,2}.h}}}{b}_{j}{\psi }^{{\overline{\varvec{\theta }}}_{\varvec{j}}}$$

Where now $${\overline{\varvec{\theta }}}_{\varvec{j}} \,runs\, from\, 1\, to \,u+\,size\, of\, \overline{\varvec{\theta }}\left(G\right)$$

We now compare with Everett’s interpretation.

We note that for $\overline{\varvec{\theta }}$ there is no longer any independent state of the observers ${O}_{{B}_{\text{1,2}.h}}\left(\overline{\varvec{\theta }}\right)$ or the $\overline{\varvec{\theta }}\left(G+1\right)+\overline{\varvec{\theta }}$. However each element of the superposition, ${\varphi }_{i}^{{O}_{{B}_{\text{1,2}.h}}}{\psi }^{{\overline{\varvec{\theta }}}_{\varvec{j}}}$, is in a particular eigenstate of $\overline{\varvec{\theta }}$, ${\psi }^{{\overline{\varvec{\theta }}}_{\varvec{j}}},$ and furthermore the $\overline{\varvec{\theta }}$ - ${O}_{{B}_{\text{1,2}.h}}\left(\overline{\varvec{\theta }}\right)$ state, ${\varphi }_{i}^{{O}_{{B}_{\text{1,2}.h}}}{\psi }^{{\overline{\varvec{\theta }}}_{\varvec{j}}}$, describes all observers at ${\overline{\varvec{\theta }}}_{\varvec{j}}$ as definitely perceiving that particular system state.

Please compare with Everett’s thesis^39^:

- We note that there is no longer any independent system state or observer state, although the two have become correlated in a one-one manner. How- ever, in each element of the superposition (2.3), if ${{\upvarphi }}_{\text{i}}{{\uppsi }}_{\text{i}[\dots .,{{\upalpha }}_{\text{i}}]}^{\text{O}}$, the object-system state is a particular eigenstate of the observer, and furthermore the observer-system state describes the observer as definitely perceiving that particular system state. It is this correlation which allows one to maintain the interpretation that a measurement has been performed.

We note that for each observer at the superposition combination ${\varphi }_{i}^{{O}_{{B}_{\text{1,2}.h}}}{\psi }^{{\overline{\varvec{\theta }}}_{\varvec{j}}}$ the encoded eigenvalue ${\alpha }_{\varvec{i}}$ of ${\varphi }_{i}^{{O}_{{B}_{\text{1,2}.h}}}$ is encoded in ${\psi }^{{\overline{\varvec{\theta }}}_{\varvec{j}}}$ subjectively the same as in the ${\psi }_{i[\dots .,{\alpha }_{i}]}^{O}$ of Everett’s. In contrast to Everett the memory $\left[\dots .{\alpha }_{\varvec{i}}\right]$ is not constant but subjectively changes so we should note it as in the next measurements as $\left[\dots .{\alpha }_{\varvec{i}}^{{{\prime \prime}}}.\right]$.

In this formalism ${\varphi }_{i}^{{O}_{{B}_{\text{1,2}.h}}}$ Is the objective property transformation to the subjective measurement thus while ${\psi }^{{\overline{\varvec{\theta }}}_{\varvec{j}}}$ is the purely subjective knowledge of an observer of the universe. Each world, in the MWI meaning, is a world line of objective observations in superposition with an observer subjective wave function. We can identify the world line as the ${\varphi }_{i}^{{O}_{{B}_{\text{1,2}.h}}}$, ${\phi }_{i}^{{O}_{{B}_{\text{1,2}.h}}},{\eta }_{i}^{{O}_{{B}_{\text{1,2}.h}}}$ …. sequences.

Let’s define the equivalent of the MWI relative state taken from Everett’s thesis^39^:

*“We now introduce the concept of a relative state-function*,* which will play a central role in our interpretation of pure wave mechanics. Consider a composite system* ${S=S}_{1}$ *+* ${S}_{2}$ *in the state* ${\psi }^{\varvec{S}}$. *To every state* $\eta$ *of* ${S}_{2}$ *we associate a state of* ${S}_{1}$, ${\psi }_{rel}^{\eta }$, *called the relative state in* ${S}_{1}$ *for* $\eta$ *in* ${S}_{2}$ *through: Definition.*

2.9 $${\psi }_{rel}^{\eta }=N\sum _{i}{(\varphi }_{i}\eta ,{\psi }^{\varvec{S}}) {\varphi }_{i}^{\prime \prime}$$

So, we have for the one ${O}_{{B}_{k}}$ and single ${\psi }^{{\varvec{\theta }}_{\varvec{j}}}$ we decompose ${\psi }^{{\varvec{\theta }}_{\varvec{j}}}$ into it’s eigenfunctions ${\phi }_{k}$

2.10 $${\psi }^{{{\varvec{\theta }}_{\varvec{j}}(\varvec{M}+1)+O}_{{B}_{k}}}=\sum _{i}{{a}_{i}\varphi }_{i}^{{O}_{{B}_{k}}}{\psi }^{{\varvec{\theta }}_{\varvec{j}}}=\sum _{k}\sum _{i}{{a}_{i}\varphi }_{i}^{{O}_{{B}_{k}}}{{c}_{k}\phi }_{k}$$

$${\varphi }_{i}^{{O}_{{B}_{k}}}{\phi }_{k}$$

2.11 $${\psi }^{{\varvec{\theta }(\varvec{M}+1)+O}_{{B}_{k}} }=\sum _{j}\sum _{i}{{a}_{i}\varphi }_{i}^{{O}_{{B}_{k}}}{b}_{j}{\psi }^{{\varvec{\theta }}_{\varvec{j}}}=\sum _{j}\sum _{k}\sum _{i}{{a}_{i}\varphi }_{i}^{{O}_{{B}_{k}}}{{{b}_{j}c}_{{k}_{j}}\phi }_{{k}_{j}}$$

So, the relative state in ${O}_{{B}_{k}}$ for ${\varvec{\theta }}_{\varvec{j}}$ is

2.12 $${\psi }_{rel}^{{\varvec{\theta }}_{\varvec{j}}}=\frac{1}{Z}\sum _{i}\sum _{k}{(\varphi }_{i}^{{O}_{{B}_{k}}}{{c}_{{k}_{j}}\phi }_{{k}_{j}},{\psi }^{{\varvec{\theta }(\varvec{M}+1)+O}_{{B}_{k}} }){\varphi }_{i}^{{O}_{{B}_{k}}}$$

Where $Z$ is a normalization constant. Thus ${\psi }_{rel}^{{\varvec{\theta }}_{\varvec{j}}}$ correctly gives the conditional expectation of all operators in ${\psi }^{{O}_{{B}_{k}}}$ conditioned by the state ${\psi }^{{\varvec{\theta }}_{\varvec{j}}}$ in ${\psi }^{\varvec{\theta }(\varvec{M}+1)}$

The relative state in $\varvec{\theta }$ for ${\varphi }_{i}^{{O}_{{B}_{k}}}$ is

2.13 $${\psi }_{rel}^{{\varphi }_{i}^{{O}_{{B}_{k}}}}=\frac{1}{Z}\sum _{j}\sum _{k}{(\varphi }_{i}^{{O}_{{B}_{k}}}{{c}_{{k}_{j}}\phi }_{{k}_{j}},{\psi }^{{\varvec{\theta }(\varvec{M}+1)+O}_{{B}_{k}} }){{c}_{{k}_{j}}\phi }_{{k}_{j}}$$

Where $Z$ is a normalization constant. Thus ${\psi }_{rel}^{{\varphi }_{i}^{{O}_{{B}_{k}}}}$ correctly gives the conditional expectation of all operators in ${\psi }^{\varvec{\theta }(\varvec{M}+1)}$ conditioned by the state ${\varphi }_{i}^{{O}_{{B}_{k}}}$ in ${O}_{{B}_{k}}$.

### Coupling to Rovelli’s relational quantum mechanics

It’s important to highlight that DHT does not fall within the confines of either the quantum or classical paradigms. Both these paradigms naturally emerge from the p-adic relational tree, as demonstrated in^1–3,40^, without the need for any additional assumptions other than the acceptance of the Leibniz Principle. The main idea behind RQM is that different observers may upon measurement/interaction give different but equally accurate accounts of the same system. This idea is equivalent to the subjective wave function outlined above as well as the fact that an event newly measured will generally have different relations with all previous events thus two observers will generally have generally different subjective knowledge on the same event. More over, in accordance with RQM, while deriving the Emergent many worlds interpretation in the relational information framework it is evident That the notion of *state* is inherently relative to a particular observer. There is no privileged, “real” description of a state that is observer-independent. Thus, the state vector is not an absolute representation but rather a description of the correlation between certain degrees of freedom within the observer and the observed system.

Notably, *Rovelli’s Relational Quantum Mechanics* (RQM) shares a significant ideological similarity with DHT^41^, particularly in their treatment of quantum phenomena. However, RQM posits that all systems are inherently quantum systems. Like DHT, RQM leverages the concept that any quantum mechanical measurement can be deconstructed into a series of yes-no questions, which is then used to formulate the state of a quantum system (relative to a given observer, much like in DHT).

In contrast to DHT, RQM asserts the completeness of quantum mechanics. Accordingly, RQM posits that there are no hidden variables or additional factors that need to be introduced into quantum mechanics, based on current experimental evidence. As demonstrated in^5^, quantum theory can be viewed as an emerging theory stemming from a relational structure. Consequently, notions such as completeness and hidden variables become irrelevant. From this perspective, the various interpretations of quantum mechanics can be seen as corresponding to different emergence frameworks for quantum theory from an event-based relational structure.

We compare the postulates of RQM with the consequences of DHT and show they align. Originally RQM included two empirical postulates:

- *Postulate 1* Maximum Extractable Information: RQM posits that there exists a maximum amount of relevant information that can be extracted from a quantum system. In DHT, this aligns with the assertion that the maximal information about an event is encoded within the event branch contained in the infinite p-adic tree.
- *Postulate 2* Continuous Information Extraction: According to RQM, it is always possible to obtain new information from a system. Similarly, DHT acknowledges that by introducing more events or asking more questions within the relational structure, additional information is added to each event’s relations with other events. This process is mathematically described as adding and elongating branches of a dendrogramic tree or adding nodes, or “questions,” to the initial configuration of nodes.

Recently, additional postulates were introduced in the RQM interpretation^42^:

1. Relative facts: Events, or facts, can happen relative to any physical system. In our model framework the subjectivity of information acquired by an observer fulfils this postulate more over ${\psi }^{{O}_{{B}_{k}}}$ which is the objective wave function, or property of the observer is only a relative concept to another observer.
2. No hidden variables: Unitary quantum mechanics is complete. As demonstrated in^5^ and in the current model, quantum theory can be seen as emerging from a relational structure. Consequently, concepts like completeness and hidden variables become irrelevant.
3. Relations are intrinsic: The relation between any two systems A and B is independent of anything that happens outside these systems’ perspectives. As is shown in the current model interaction/measurements between observers or group of observers pertains only to the relational information one observer (group of observers) acquire on the other (another group). In that sense also the “objective” ${\psi }^{{O}_{{B}_{k}}}$ is dynamically evolving only in relation to another observer or group of observers.
4. Relativity of comparisons: It is meaningless to compare the accounts relative to any two systems except by invoking a third system relative to which the comparison is made. In our model, each observer possesses a description of the scenario that is accurate from their perspective. However, due to the relativity of comparisons, these descriptions cannot be meaningfully compared. This parallels the situation described in Wigner’s friend case, as elucidated by Rovelli regarding the significance of the aforementioned postulate.
5. Measurement: An interaction between two systems results in a correlation within the interactions between these two systems and a third one; that is, with respect to a third system W, the interaction between the two systems S and F is described by a unitary evolution that potentially entangles the quantum states of S and F. In our model observer S and F are measured by W their eigenfunctions are both now part of its world line and evolve in full correlation to the worldline trajectory of W.
6. Internally consistent descriptions: In a scenario where F measures S, and W also measures S in the same basis, and W then interacts with F to “check the reading” of a pointer variable (i.e., by measuring F in the appropriate “pointer basis”), the two values found are in agreement. In our model, two “same readings” correspond to the same eigenfunctions in the observer’s world line, which results in no movement of the observer through the θ parameter space upon the second checking. Consequently, interaction without movement leads to the same pointer.

Please notice again that all postulates of RQM are emergent in the relational information framework.

For more details on DHT coupling to Smolin’s approach to quantum theory emergence, see Appendix A2.3 and^43–46^.

## Non-ergodicity of the relational information framework

In this section we will show that the relational information framework is generally non-ergodic.

Let us choose a level $\mathbf{N}$ and another level n such that $\mathbf{N}>\mathbf{n}$ and $\varvec{m}\varvec{o}\varvec{d}\left(\mathbf{N},\mathbf{n}\right)=0$. The N’th level is all parameter points ${\varvec{\theta }}_{\varvec{N}}$ in the Minkowski-like space that represent dendrograms with N edges/different events. We know that a single dendrogram is a certain configuration of $\varvec{M}$ nodes. Moreover, we know we can represent it as a union of all $$\left(\begin{array}{c}\varvec{M}\\ \varvec{n}\end{array}\right)$$ combinations of partial configurations of n nodes that are contained in the M configuration. Each of the $$\left(\begin{array}{c}\varvec{M}\\ \varvec{n}\end{array}\right)$$ unique configuration can be mapped to the n-level parameter points ${\varvec{\theta }}_{\varvec{n}}$ in the minkowski-like space that represent dendrograms with n edges/different events. Let’s consider all the N’th level ${\varvec{\theta }}_{{\textbf{N}}^{\prime }}\varvec{s}$ points representing all unique dendrograms at the N’th level. If there are *R* ${\varvec{\theta }}_{\textbf{N}}$ parameter points we have *R* unique configuration of ${\varvec{M}}_{\varvec{i}}$ nodes at level *N.* Again each of the R node configurations (*M1*,*M2*,*M3*….*M*_*R*_.) is a union of all $$\left(\begin{array}{c}{\varvec{M}}_{\varvec{i}}\\ \varvec{n}\end{array}\right),\ \varvec{i}=\text{1,2}...\varvec{R}$$. combinations of partial configurations of n nodes that are contained in the M configuration. Then again, each of the $$\left(\begin{array}{c}{\varvec{M}}_{\varvec{i}}\\ \varvec{n}\end{array}\right)$$ unique configuration can be mapped to the n-level parameter points ${\varvec{\theta }}_{\varvec{n}}$ in the minkowski-like space that represent dendrograms with n edges/different events. Let’s denote ${\varvec{a}}_{\varvec{j}}\in \varvec{a}$ as a unique configuration from the set of all $$\left(\begin{array}{c}{\varvec{M}}_{\varvec{i}}\\ \varvec{n}\end{array}\right),\ \varvec{i}=\text{1,2}...\varvec{R}$$, configurations for all $\varvec{i}.$ we can have a discrete distribution of all unique configurations $\varvec{f}\left({\varvec{a}}_{\varvec{j}}\right)={\varvec{p}}_{\varvec{j}}$. Similarly for a single point in ${\varvec{\theta }}_{\varvec{N}}$ we have ${\varvec{b}}_{\varvec{k}}\in \varvec{b}\, \varvec{such}\, \varvec{that}\, \varvec{b}\complement\varvec{a}$ as a unique configuration from the set of all $$\left(\begin{array}{c}{\varvec{M}}_{\varvec{i}}\\ \varvec{n}\end{array}\right)$$ where *i* is fixed. Thus, for a single point we have a uniform, trivial, discrete distribution $$\varvec{f}\left({\varvec{b}}_{\varvec{k}}\right)={\varvec{p}}_{\varvec{k}}=1/\left(\begin{array}{c}{\varvec{M}}_{\varvec{i}}\\ \varvec{n}\end{array}\right)$$.

Let’s now consider an observer that measures some N events over time. Thus, we have an ordered sequence E= {E1, E2, E3…EN}. Dividing the sequence to N/n blocks and constructing from each block a n-edge dendrogram is equivalent to take have some $\varvec{g}$ ordered sequence of the $\varvec{b}$ configurations but generally $\varvec{g} \complement \varvec{b}$ so we have a configuration N/n unique configuration ${\varvec{b}}_{\varvec{r}} \varvec{r}=\text{1,2}\dots \left(\frac{\mathbf{N}}{\mathbf{n}}\right).$ Again, we obtain, for a single observer, $\varvec{f}\left({\varvec{b}}_{\varvec{r}}\right)={\check{\varvec{p}}}_{\varvec{r}}=1/\left(\frac{\varvec{N}}{\varvec{n}}\right)$.

Let’s prove that the mean of $f\left({b}_{r}\right)\ne f\left({a}_{j}\right), r=\text{1,2}\dots \left(\frac{\text{N}}{\text{n}}\right)\,and\, {b}_{r}\complement a.$ Since all ${a}_{j}$ represent unique dendrogram lets denote them with a discreate values ${a}_{j}\in \text{{N}}.$ First let’s order ${p}_{j}$ such that ${p}_{1}\ge {p}_{2}\ge {p}_{3\dots }\ge {p}_{T}\ge 0$ and accordingly denote each ${a}_{j}$ such that

${a}_{1}>{a}_{2}>{a}_{3\dots }>{a}_{T}>0$ as a consequence, we have for the observer ${b}_{1}>{b}_{2}>{b}_{3\dots }>{b}_{\frac{\varvec{N}}{\varvec{n}}}>0$

And since ${\check{p}}_{1}={\check{p}}_{2}={\check{p}}_{3}\dots {\check{p}}_{\frac{\varvec{N}}{\varvec{n}}}=\frac{\varvec{n}}{\varvec{N}}$ both pairs of series fulfill Chebyshev sum inequality:

$$\frac{1}{T}\sum _{i=1}^{T}{a}_{i}{p}_{i}\ge \left(\frac{1}{T}\sum _{i=1}^{T}{a}_{i}\right)\left(\frac{1}{T}\sum _{i=1}^{T}{p}_{i}\right)$$

3.1 $$\frac{N}{n}\sum _{j=1}^{\frac{N}{n}}{b}_{j}{\check{p}}_{j}\ge \left(\frac{n}{N}\sum _{j=1}^{\frac{N}{n}}{b}_{j}\right)\left(\frac{n}{N}\sum _{j=1}^{\frac{N}{n}}{\check{p}}_{j}\right)$$

Thus:

$$\sum _{i=1}^{T}{a}_{i}{p}_{i}-\left(\frac{1}{T}\sum _{i=1}^{T}{a}_{i}\right)\ge 0 since \left(\frac{1}{T}\sum _{i=1}^{T}{p}_{i}\right)= \frac{1}{T}$$

3.2 $$\sum _{j=1}^{\frac{N}{n}}{b}_{j}{\check{p}}_{j}-\left(\frac{n}{N}\sum _{j=1}^{\frac{N}{n}}{b}_{j}\right)\ge 0 since \left(\frac{n}{N}\sum _{j=1}^{\frac{N}{n}}{\check{p}}_{j}\right)=\frac{n}{N}$$

Without loss of generality:

3.3 $$\sum _{i=1}^{T}{a}_{i}{p}_{i}-\left(\frac{1}{T}\sum _{i=1}^{T}{a}_{i}\right)\ge \sum _{j=1}^{\frac{N}{n}}{b}_{j}{\check{p}}_{j}-\left(\frac{n}{N}\sum _{j=1}^{\frac{N}{n}}{b}_{j}\right)$$

Thus:

$$\sum _{i=1}^{T}{a}_{i}{p}_{i}-\sum _{j=1}^{\frac{N}{n}}{b}_{j}{\check{p}}_{j}\ge \left(\frac{1}{T}\sum _{i=1}^{T}{a}_{i}\right)-\left(\frac{n}{N}\sum _{j=1}^{\frac{N}{n}}{b}_{j}\right)\, we\, are\, done$$

If

$$\left(\frac{1}{T}\sum _{i=1}^{T}{a}_{i}\right)-\left(\frac{n}{N}\sum _{j=1}^{\frac{N}{n}}{b}_{j}\right)>0\, we\, are\, done$$

else

3.4 $$\left(\frac{1}{T}\sum _{i=1}^{T}{a}_{i}\right)-\left(\frac{n}{N}\sum _{j=1}^{\frac{N}{n}}{b}_{j}\right)\le 0,$$

resulting in either

$$\sum _{i=1}^{T}{a}_{i}{p}_{i}-\sum _{j=1}^{\frac{N}{n}}{b}_{i}{\check{p}}_{j}>0 , \sum _{i=1}^{T}{a}_{i}{p}_{i}-\sum _{j=1}^{\frac{N}{n}}{b}_{i}{\check{p}}_{j}<0 \text{o}\text{r} \sum _{i=1}^{T}{a}_{i}{p}_{i}-\sum _{j=1}^{\frac{N}{n}}{b}_{i}{\check{p}}_{j}=0$$

With the two first options we are done, Else if the equality holds

$$\sum _{i=1}^{T}{a}_{i}{p}_{i}-\sum _{j=1}^{\frac{N}{n}}{b}_{i}{\check{p}}_{j}=0\ge \left(\frac{1}{T}\sum _{i=1}^{T}{a}_{i}\right)-\left(\frac{n}{N}\sum _{j=1}^{\frac{N}{n}}{b}_{j}\right)$$

But $\left(\frac{n}{N}\sum _{j=1}^{\frac{N}{n}}{b}_{j}\right)$ = $\sum _{j=1}^{\frac{N}{n}}{b}_{i}{\check{p}}_{j}$ thus the following should hold: $\sum _{i=1}^{T}{a}_{i}{p}_{i}=\frac{n}{N}\sum _{j=1}^{\frac{N}{n}}{b}_{j}\ge \left(\frac{1}{T}\sum _{i=1}^{T}{a}_{i}\right)$

$$\sum _{i=1}^{T}{a}_{i}-\frac{Tn}{N}\sum _{j=1}^{\frac{N}{n}}{b}_{j}=z. z\le 0$$

3.5 $$\sum _{i=1}^{T}{(a}_{i}+\left|z\right|/T)-\frac{Tn}{N}\sum _{j=1}^{\frac{N}{n}}{b}_{j}=0$$

but we didn’t set ${a}_{i}$ and since they represent unique node configurations, we can give them any discrete values so let as “name” them: ${a}_{i}=(group\, size-i)*100$ then we change only one of the ${a}_{i}^{{\prime }}s,$ one that is not contained in $b$ to a value

$${if z<0 a}_{i}=(group\, size-i)+\frac{q*\left|z\right|}{T}, {a}_{i}\notin b, q\ne 0$$

$$if z=0 {a}_{i}=(group\, size-i)+\frac{q*1}{T} \,and \,q \,is\, such\, that$$

$\left(group\, size-i-1\right)*100<{a}_{i}<\left(group\, size-i+1\right)*100 \,and\, {a}_{i}\ne i*100$ will result in

$$\sum _{i=1}^{T}{(a}_{i}+\left|z\right|/T)-\frac{Tn}{N}\sum _{j=1}^{\frac{N}{n}}{b}_{j}=\frac{q*\left|z\right|}{T}\ne 0$$

3.6 $$\sum _{i=1}^{T}{a}_{i}{p}_{i}-\sum _{j=1}^{\frac{N}{n}}{b}_{i}{\check{p}}_{j}=0\ge \left(\frac{1}{T}\sum _{i=1}^{T}{a}_{i}\right)-\left(\frac{n}{N}\sum _{j=1}^{\frac{N}{n}}{b}_{j}\right)>0.$$

Which is a contradiction.

Thus at least for the above “naming” procedure we have more possibilities to “name” the configuration such that

$$|\sum _{i=1}^{T}{a}_{i}{p}_{i}-\sum _{j=1}^{\frac{N}{n}}{b}_{j}{\check{p}}_{j}|>0$$

Let’s assign for each unique dendrogram ${c}_{v}$ at the n level all its unique configurations ${a}_{i}$ that map to it.

$$f\left(\{{a}_{h}{\}}_{H}\right)={c}_{i}$$

$$H\in all\, {a}^{{\prime }}s\, such \,that\, {a}_{h} \,maps\, to\, {c}_{i} \,and \,does \,not\, map \,to\, {c}_{j}\, for\, i\ne j$$

Thus, group $H$ is $L$ size ascending combination of 1, 2,… $T$

Again: ${p}_{{h}_{1}}\ge {p}_{{h}_{2}}\ge {p}_{{h}_{3}\dots }\ge {p}_{{h}_{L}}\ge 0$ and accordingly denote each ${a}_{j}$ such that

$${a}_{{h}_{1}}>{a}_{{h}_{2}}>{a}_{{h}_{3}}\dots .>{a}_{{a}_{{h}_{L}}}>0$$ resulting in

$$\frac{1}{L}\sum _{i=1}^{L}{a}_{{h}_{i}}{p}_{{h}_{i}}\ge (\frac{1}{L}\sum _{i=1}^{L}{a}_{{h}_{i}}\left)\right(\frac{1}{L}\sum _{i=1}^{L}{p}_{{h}_{i}})$$

$$\sum _{i=1}^{L}{a}_{{h}_{i}}{p}_{{h}_{i}}\ge (\frac{1}{L}\sum _{i=1}^{L}{a}_{{h}_{i}}\left)\right(\sum _{i=1}^{L}{p}_{{h}_{i}})$$

$$where {\dddot{p}}_{j}=(\sum _{i=1}^{L}{p}_{{h}_{i}})<1$$

But $\sum _{i}^{L}{a}_{{h}_{i}}{p}_{{h}_{i}}={\dddot{p}}_{j}{c}_{j}$ where ${\dddot{p}}_{j}$ is the ${c}_{j}$ fraction of all the unique ${a}_{{h}_{i}}$ configurations that map to ${c}_{j}$. Thus

$${\dddot{p}}_{j}{c}_{j}\ge (\frac{1}{L}\sum _{i}^{L}{a}_{{h}_{i}}\left)\right(\sum _{i}^{L}{p}_{{h}_{i}})$$

And we set

$$\sum _{j=1}^{K}{\dddot{p}}_{j}{c}_{j}=\frac{1}{K}\sum _{j=1}^{K}\sum _{{H}_{j}}{a}_{{h}_{i}}{p}_{{h}_{i}}$$

For a single observer we again repeat as above:

$$U\in all \,{b}^{{\prime }}s\, such\, that\, {b}_{u} \,maps\, to\, {c}_{i}\, and \,does\, not\, map \,to\, {c}_{j} \,for\, i\ne j$$

Thus, the group $U$ is some $L1$ size ascending combination of 1, 2,… $N/n$

Again: as all ${\check{p}}_{j}$ are equal then ${\check{p}}_{{u}_{1}}={\check{p}}_{{u}_{1}}={\check{p}}_{{u}_{1}}\dots .={\check{p}}_{{u}_{L1}}=n/N$ of the group U1 and accordingly denote each ${{b}_{u}}_{j}$ such that

$${{b}_{u}}_{1}>{{b}_{u}}_{2}>{{b}_{u}}_{3}\dots .>{{b}_{u}}_{L1}>0$$ resulting in

$$\sum _{j=1}^{L1}{{b}_{u}}_{j}{\check{p}}_{{u}_{j}}={q}_{k}{c}_{k}$$

And again, since all ${b}_{1}>{b}_{2}>{b}_{3 }\dots >{b}_{N/n}>0$ and ${\check{p}}_{1}={\check{p}}_{1}={\check{p}}_{1}\dots {\check{p}}_{{1}{\frac{N}{n}}}=\frac{n}{N}$ where

$$f\left(\{{b}_{u}{\}}_{U}\right)={c}_{j} \text{with probability}\,{q}_{j}$$

$$U\in \,all\, {b}^{{\prime }}s \,such\, that \,{b}_{u}\, maps\, to\, {c}_{j} \,and\, does\, not\, map\, to\, {c}_{i} \,for\, i\ne j$$

Thus, the group $U$ is some $L1$ size ascending combination of 1, 2,… $T$

Again: we select all $\dddot{p}\ne 0$ ${\check{p}}_{{u}_{1}}={\check{p}}_{{u}_{1}}={\check{p}}_{{u}_{1}}\dots .={\check{p}}_{{u}_{L1}}=n/N$ and where we order ${b}_{{u}_{j}}$ such that

$${b}_{{u}_{1}}>{b}_{{u}_{2}}>{b}_{{u}_{3}}\dots .>{b}_{{u}_{L1}}>0$$ resulting in

$$\sum _{j=1}^{K1}{q}_{j}{c}_{j}=\sum _{i=1}^{K1}\sum _{{U}_{i}}{b}_{{u}_{j}}{\check{p}}_{{u}_{j}}=\sum _{i=1}^{K1}\frac{n}{N}\sum _{{U}_{i}}{b}_{{u}_{j}}$$

For all ${\varvec{\theta }}_{\varvec{N}}$ points we have already shown in steps (4.1–4.6) we have much more “naming” procedure of ${a}_{i}$ that

$$\sum _{j=1}^{K}{\dddot{p}}_{j}{c}_{j}-\sum _{j=1}^{K1}{q}_{j}{c}_{j}=\sum _{j=1}^{K}\sum _{{H}_{j}}{a}_{{h}_{i}}{p}_{{h}_{i}}\sum _{j=1}^{K1}\frac{n}{N}\sum _{{U}_{j}}{b}_{{u}_{i}}=\sum _{i=1}^{T}{a}_{i}{p}_{i}-\sum _{j=1}^{\frac{N}{n}}{b}_{j}{\check{p}}_{j}>0$$

And thus by “naming” correctly the ${a}_{i}{\prime }s$ in ${\theta }_{N}$ the observer mean will be different also at ${\theta }_{n}$

**Fig. 5:**
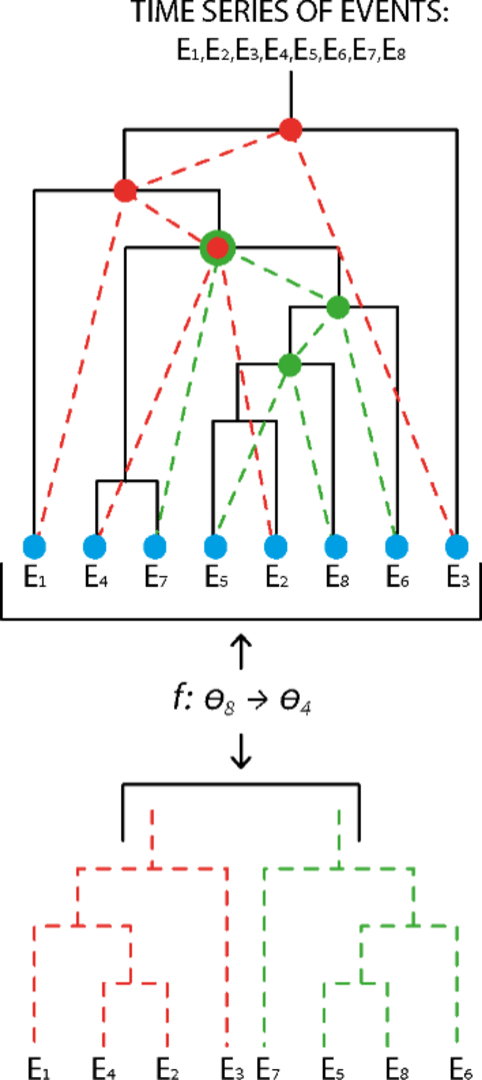
Illustration of the consequence of decomposing a time series of events into blocks of events for relational information structures. Construction of the relational information structure from a discrete consecutive series of events. By decomposing the series of events into two blocks {E_1_ E_2_ E_3_ E_4_} and {E_5_ E_6_ E_7_ E_8_} and constructing from each block a dendrogram is equivalent to applying the map $f: {\theta }_{N}\to {\theta }_{n}$ to two partial node (“question”) configurations.

Non-ergodicity has been suggested as causing non-locality^47^, furthermore numerical simulations of the non-ergodic series of block dendrograms have demonstrated apparent non-locality, with correlations values that violate the CHSH inequality (see^2^ and Supplementary Appendix A3.1 details for two such simulations setups). The process in^2^ involves Alice and Bob selecting “different naming,” and confirming if that naming aligns with the observed block dendrogram. We extend, in the current study, the “naming” concept to any type of naming, Enabled by the distinct parameterization of dendrogramic structures in a Minkowski-like space. Additionally, numerical simulations^1^ of such mapping ( $f: {\varvec{\theta }}_{\varvec{N}}\to {\varvec{\theta }}_{\varvec{n}}$ ) resulting in less distinguishability which in turn explains explicate-implicate order with reminiscent correlations between the ${a}_{i}^{{\prime }}s\, and\, {c}_{i}{\prime }s$ configurations (Fig. 5). We note that the implicate and explicate order are an ontic and epistemic ideas established by Bohm which emerge both in DHT with simple and clear connection.

## Discussion

Exploring the relational information framework with the p-adic treelike geometry offers new insights on relational information evolution. Experimental data, represented by dendrograms, undergo dynamic restructuring upon the addition of new information. By connecting relational event models with conventional ones based on real spacetime, we introduce a statistical causality approach, encoding dendrograms with real parameters to map them onto four-dimensional spacetime. These parameter spaces, reflecting subjective observer knowledge, enable transformations akin to special relativity within the dendrogramic configuration space, expanding the scope of DHT.

The intriguing co-existence of s Bohmian mechanics with the Many-Worlds Interpretation within the framework of relational information using DHT, along with the fulfillment of Rovelli’s RQM postulates, underscores the fundamentality of the relational approach. Our model consolidates the dynamics between observers and systems into what we term an “observers universe,” where measurements are conducted by observers on observers themselves.

An interesting feature of our model is the tight link between an observer’s measurements and their world line, underscoring subjectivity’s role in driving dynamics. While the ontic world line remains static, represented by the p-adic infinite tree, dynamics emerge from subjective parameters, revealing subjectivity as the primary driver of dynamics.

The adherence to the ontic/epistemic Leibniz principle directly leads to Machian relationism, not as an assumption, in contrast to theories like shape dynamics and Brans-Dicke theory^28,29,32^, but as an intrinsic outcome of the p-adic tree representation of events. Furthermore, the endorsement of the Leibniz principle gives rise to a background-independent theory, akin to theories such as shape dynamics, loop quantum gravity, spin foams, and causal set theory^28–31,48^. We note that Rovellis *partial observables* which are quantities that can be measured locally, but don’t provide complete information by themselves are in DHT the events themselves. In other words, a partial observable is any quantity you could theoretically measure, but which lacks meaning in isolation. These observables are not gauge-invariant and are Context-dependent. A *complete observable* was defined by Rovelli as an expression that combines partial observables in a way that results in a gauge-invariant quantity. Complete observables are constructed by linking two or more partial observables to create a *relational* observable. Thus the partial observables are the objective but since an observer in DHT has a subjective view of the universe the single event only have meaning when related to other event. Therefore, each dendrogram, which is in fact some configuration of Leibnitz monads, is already describing all observables as *relational* observable. The basic relational and dynamical laws of these dendrograms suggests (although still needs to be proved) gauge invariance on the Minkowski-like parameter space - implying a correspondence to Rovelli’s complete observables.

DHT extends beyond physics, applied in brain modeling, medical diagnostics, and potentially machine learning. Our approach aids in understanding clinical data analysis, enriching our understanding of complex systems^7,8^.

Overall, studies within DHT hint at unifying quantum and classical paradigms, yet more research is required to understand how phenomena like gravitation and conventional physics emerge from this framework.

## Electronic supplementary material

Below is the link to the electronic supplementary material.

Supplementary Material 1

## Data Availability

The datasets generated and/or analysed during the current study are available in the kaggle repository, https://www.kaggle.com/datasets/odedshor/dynamics-of-relational-information.
