# Supplementary material for: Relational information framework, causality, unification of quantum interpretations and return to realism through non-ergodicity
Source: Sci Rep. 2025 Mar 10;15:8170. doi: 10.1038/s41598-025-90225-7 (PMC11891336; doi:10.1038/s41598-025-90225-7)
Supplement: Supplementary file 1 — Supplementary Material 1 [file 41598_2025_90225_MOESM1_ESM.pdf]

Appendix

A1. Observer-dependent subjective knowledge of universe

In DHT, it is evident that two or more observers can hold different epistemic perspectives of the universe they observe, essentially having different relational structures (p-adic trees representation of relational information) . Conversely, two or more observers can also share identical epistemic views of the universe, even if they have observed different events, indicating that these observers possess equivalent inter-relations among the events they have measured. In this context, it becomes clear that an observer invariably maintains a subjective view of the universe, irrespective of whether the universe is of a classical or quantum nature. This notion harmonizes with *Bohr's principle of complementarity and the concept of event physics*. Bohr consistently emphasized that the outcomes of physical observables are not objective properties of systems but are generated within the process of measurement. Similarly, the observer-dependent perspective of the universe aligns with the principles of special relativity, where the measurement of an event in spacetime is contingent on the observer's momentary inertial frame.

When we define an observer by their worldline curve, whether undergoing acceleration or not, against the background of spacetime, each observer will inevitably derive a distinct ontic relational perspective of the universe. It is crucial to emphasize that two different observers sharing the same worldline are not admissible, as they would essentially be one and the same observer, in accordance with Leibniz's principle. This unique perspective is contingent upon the information pertaining to events, situated within the spacetime background, and its transmission to the observer, whether they are in motion with acceleration or moving at a constant velocity within spacetime.

Therefore, within the DHT framework, epistemic relational information regarding the universe is inherently observer-dependent. In this context, measurements can be considered "subjective" in the quantum mechanics sense and/or observer-dependent as per the principles of special relativity, but both ultimately contribute to a subjective epistemic knowledge of the universe.

A1.1 Real parametrization of dendrograms

In our study, we employed the following equation to facilitate our analysis:

The representation of a dendrogram branch, denoted as $edge_i$, can be expressed as the sum of a series:

$edge_i = \sum_{j=0}^{k} a_j \times p^j, \quad a_j = 0,1..p-1.$ (1.1*)*

Throughout this study we will us p=2 thus $a_j = 0,1$

where each term corresponds to the contribution of a specific level in the dendrogram's hierarchical structure. Here*,* a_j represents the binary digit at position j, with possible values of 0 or 1.

To further enhance our analysis, we introduce the concept of the monna map conversion. The monna map conversion of an event, denoted as $event_i$ *,* is computed using the formula:

$event_i = \sum_{j=0}^{k} a_j \times p^{-j-1}, \; a_j = 0,1..p-1.$ (1.2)

Throughout this study we will us p=2 thus $a_j = 0,1$

where a_j represents the binary digits (0 or 1) in the 2-adic expansion of the dendrogram branch, and k is the maximum ball level of the dendrogram.

By applying this Monna map conversion, we represent events as rational numbers on the continuous interval [0, 1]. This conversion preserves the precise relations between events, ensuring that the inherent structure and ordering within the dendrogram branches are maintained. To quantify the differences between events, we introduced the metric q_{ik}*,* which represents the absolute difference between the monna map conversions of two events*,*

we defined 5 elementary/fundamental parameters of a dendrogram

We define our dendrogramic vector, D , as follows:

$$E = event_i\,, i = 1,2 \ldots n = number\ of\ events$$
$$B = 2^{-maximal\ ball\ level\ of\ the\ dendrogram}$$
$$D = [E\ B]\ with\ elements\ D_i\,, i = 2,3 \ldots n+1$$

$V_D = (\sum_{i=0}^{k} D_i)^z$

$U_D = (\sum_{i=0}^{k} \frac{1}{D_i+1})^{z1}$

$M_D = (\sum_{i=1}^{k-1} \sum_{j=i+1}^{k} D_i \cdot D_j)^{z2}$

$R_D = (\sum_{i=1}^{k-1} \sum_{j=i+1}^{k} D_i - D_j)^{z3} = (\sum_{i=1}^{k-1} \sum_{j=i+1}^{k} q_{ij}) + \sum_{j=i+1}^{k} \left|B - D_j\right|)^{z3}$

$$r_D = (\sum_{i=1}^{k-1}\sum_{j=i+1}^{k} 1/((D_i - D_j) + 1))^{z4} =$$

$(\sum_{i=1}^{k-1}\sum_{j=i+1}^{k} 1/(q_{ij} + 1) + \sum_{j=i+1}^{k} 1/(|B - D_j| + 1))^{z4}$

$k = number\ of\ branches\ and\ thus\ events\ in\ dendrogram$ (1.3)

Where z,z1,z2,z3 and z4 each takes a random a value, in our numerical simulations *z,z1,z2,z3 and z4* (presented in section 6) will have, randomly, a value from *T=[-2 -1 -0.5 0.5 1 2]*

We then constructed from a combination of them another 55 parameters in the following way.

1. all possible two elementary parameters product combinations. Thus adding 10 more parameters
2. all possible two elementary parameters sum combinations. Thus adding 10 more parameters
3. all possible two elementary parameters division combinations. Thus adding 20 more parameters
4. all possible three elementary parameters product combinations. Thus adding 10 more parameters
5. all possible four elementary parameters product combinations. Thus adding 5 more parameters.

Overall, we have 60 possible parameters. These 60 parameters will be used for numerical deep scan of possible "dendrogramic Minkowski causal structure of observers ensemble"

A1.2 Simulations of "observers" and the possible dendrograms they can reach

process 1 was to determine how many different, unique, dendrograms are possible in some "number of events-level n".

We followed these sub-steps : Here are the sub-steps of the given process:

1. Generate 100,000 vectors of n values (representing events) for each n = 5, 6, and 7 by Randomly selecting n values from the interval [0, 1] for each vector.
2. Compute pairwise Euclidean distances between elements in each vector.
3. Construct agglomerative hierarchical cluster trees using the "single" linkage method for each vector.
4. Identify distinct dendrogram structures for each "number of events-level n".
5. Count the number of different dendrogram structures obtained for each n.

For n=5, we found m=4 different dendrogram structures. For n=6, we obtained m=9 different dendrogram structures, and for n=7, we discovered m=21 different dendrogram structures.

process 2 we produced 100 "observers" for each of the m=4,9,21 unique and different dendrograms each in its corresponding n=5,6,7 event level.

1. randomly select n numbers from the interval [0, 1]
2. construct a dendrogram D out of the n numbers (as in steps 3 and 4 of process 1)
3. identify to which of the m different dendrograms D equals to.
4. the n random numbers defines $Observer_{ij}$ i=1,2..100 j=1,2..m
4.repeat until i=100 for all j

Thus although each of the 100 "observers" collected different n events/numbers their relational observed universe is the same.

Process 3 producing all possible dendrograms an "observer" can evolve to by edding k=1,2..6 events.

1.for each "observer" we add k random numbers (k=1-6) t times (t=4000*(1+k/2) for n=5,6. t=6000*(2+k/2) for n=7)
2. construct a dendrogram D out of the n(constant numbers of the observer) and k random numbers.

Overall, at the end, for all 100 observers we produced for all k=1,2..6 we produced 4*1600000, 9*1600000 and 21*3000000 non unique dendrograms for n=5,6,7 respectively

A1.3 Establishing dendrogram-parameters coupling via numerical simulation

Although the informational geometric Minkowski-like metric is proved analytically, in practical data analysis applications, we still face the problem of determining which two dendrograms are "time-like" and which are "space-like". For that purpose we need parameter spaces that follow the usual Minkowski space-time determinant where it must possess the light-cone characteristics. In the context of spacetime intervals spacelike intervals are characterized by $\sum \Delta X^2 > c\Delta t^2$ whereas time-like intervals are characterized by $\sum \Delta X^2 < c\Delta t^2$

The problem of encoding dendrograms using real parameters is mathematically challenging and can require a significant amount of time to find a solution. In the context of DHT- theory, where simulations have provided valuable insights, we propose a method called "numerical experimenting confirmation" to select parameters. Through an extensive numerical simulation, we demonstrate the validity of our real parametrization on the space of dendrograms. While this approach does not provide a mathematical proof of the minkowski-like determinant, the likelihood of encountering dendrograms that do not conform to our parametrization is practically negligible. As a result, we confidently propose the use of this parametrization for the extended space of dendrograms. In this technical section, we present the output and findings of our numerical simulation.

In appendix A1 we outline the numerical procedures for generating different observers with certain n=5,6,7 events encoded in their dendrograms (process 1 and 2). Each initial level n has m number of unique dendrograms (for each of the m unique dendrograms we created 100 different observers).We then investigate to which dendrograms, with n+k events where k=1,2..6 , each such observer can reach by adding to its n previous events k random events (process 3).In that way we could know for a single observer in its initial "events-level n" what are its possible dendrogram it can transfer to in each of the "events-level n+k"

In each scenario of n+k events, starting from one of the m initial n level dendrogram, we examined the set of possible unique dendrograms that could be created. This analysis allowed us to determine the distribution of these unique n+k events dendrograms that arise from any of the m's unique initial dendrogram as shown in figure **S1** for n=6.

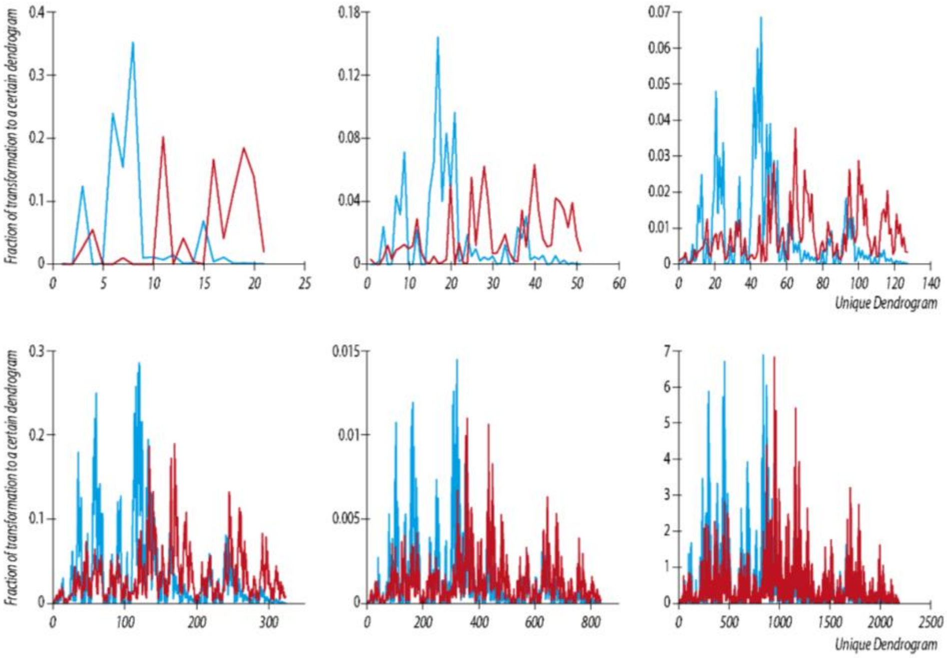

Figure S1: Distribution of Dendrograms at n+k Level for Two Different Initial Dendrograms with 6 Events. This figure illustrates the evolutionary process of two distinct initial dendrograms, namely Dendrogram A and Dendrogram B, each consisting of 6 events. The diagram showcases the distribution of dendrograms that emanate from each initial dendrogram at various n+k levels (k = 1, 2, 3, 4, 5, and 6). At each level, the initial dendrograms undergo transformation and branching, leading to the emergence of unique configurations represented by subsequent dendrogram structures. The visualization offers insights into the dynamic nature of dendrogram evolution as they progress through different levels.

Interestingly, all m dendrograms with n=6 could be transformed to all possible dendrograms at n+k levels where k has the value of 3-4. However, for the m dendrograms with n=7, this encompassing did not occurr. This implies that some of the m initial dendrograms with n=6,7 had both "space like" and "time like" as their future evolving dendrograms (figure **S2** A1-A2).

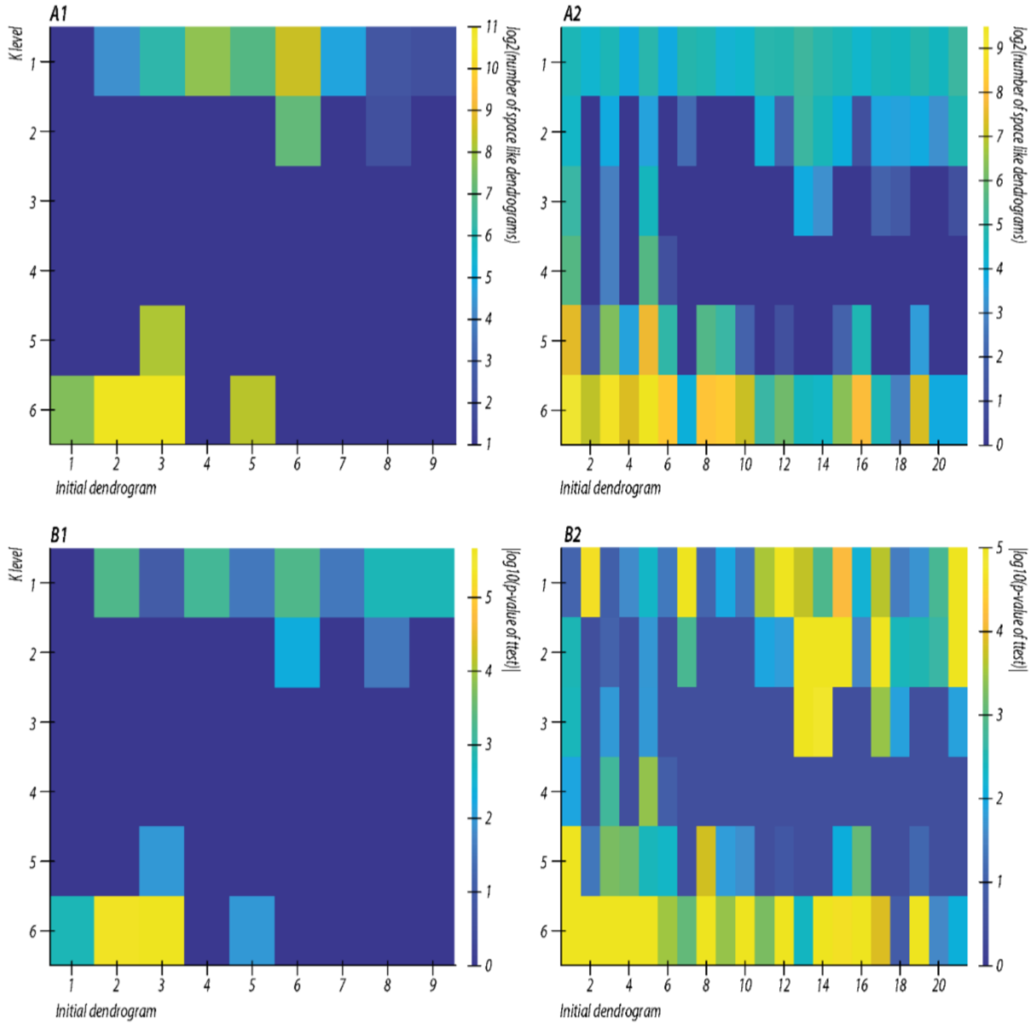

Figure S2:A1 Number of Spacelike Dendrograms for Each k (Initial Level n=6) This subfigure depicts the count of spacelike dendrograms at each k level for various initial dendrograms, all having an initial level of n=6. Each k level demonstrates a distinct number of unique spacelike dendrograms, specifically 21, 51, 127, 323, 835, and 2188, representing different configurations of dendrogram structures. **A2 Number of Spacelike Dendrograms for Each k (Initial Level n=7)** This subfigure displays the count of spacelike dendrograms at each k level for different initial dendrograms, with a shared initial level of n=7. At each k level, there are 51, 127, 323, 835, 2188, and 5798 unique spacelike dendrograms, reflecting diverse configurations of dendrogram structures. **B1 Mean Significance of Intervals in Selected Parameters of Spacelike vs. Timelike Dendrograms (Initial Level n=6)** This subfigure presents the average significance values of intervals within selected parameters for spacelike and timelike dendrograms at each k level, considering various initial dendrograms with an initial level of n=6. The comparison between spacelike and timelike dendrograms allows for an assessment of the significance variations across different intervals in the selected parameters.**B2 Mean Significance of Intervals in Selected Parameters of Spacelike vs. Timelike Dendrograms (Initial Level n=7)** This subfigure exhibits the average significance values of intervals within selected parameters for spacelike and timelike dendrograms at each k level, considering different initial dendrograms with an initial level of n=7. The comparison between spacelike and timelike dendrograms enables an examination of the significance variations across different intervals in the selected parameters.

These results raise the question of whether we can assign Minkowski-like signatures (determinant) to these dendrograms parameter spaces. We require these spaces to posess interval values opposite in sign dependent on whether two dendrograms can or cannot evolve to each other.

Interestingly, the absolute difference between some of the 60 parameter values (described in **A1**) of an initial dendrogram (one of the m possible) and its corresponding dendrograms it evolved to, at some level n+k, showed significant difference compared to the absolute difference between the parameter values an initial dendrogram (one of the m possible) and its corresponding dendrograms **it did not evolve to**, at some level n+k.

After performing 5000 iterations of random selection for z, z1, z2, z3, and z4, we identified 23 out of the 60 parameters that exhibited statistical significance ($p < 0.05$ in t-test) in at least 60% of the occurrences where “timelike” and "spacelike" dendrograms both existed for an initial specific m dendrogram. Furthermore, these parameters showed significance in at least 70% of the random selections of z, z1, z2, z3, and z4 introduced in section 3 (an example is shown in figure S2 B.1-B.2).

With those 23 parameters we examined all possible combinations such that:

$$INTERVAL_i = (\theta'_{i\,initial\,dendrogram} - \theta'_{i\,n+k\,level\,dendrogram})^2 \quad for\ i = 1,2,3$$

Where we define *n= initial dendrogram level/ number of events*

n+k=level of transmitted/not transmitted dendrograms, k=1,2,3,4,5,6.

$$INTERVAL_4 = (\sqrt{n}\theta'_{i\,initial\,dendrogram} - (\sqrt{n+k})\theta'_{i\,n+k\,level\,dendrogram})^2 \quad for\ i = 4$$

Such that the following equation :

$INterval = INTERVAL_1 + INTERVAL_2 + INTERVAL_3 - s2 * INTERVAL_4$

Will fulfill the condition that $INterval$ values for spacelike and timelike dendrograms have opposite signs

We conducted 5000 iterations, exploring various combinations of constants (z, z1, z2, z3, z4, and s2), to identify parameter sets consistently demonstrating opposite sign intervals between timelike and spacelike dendrograms. This analysis encompassed all initial m dendrograms (limited to n=6) and their corresponding spacelike/timelike dendrograms at n+k levels (k=1, 2, ..., 6).

Remarkably, our analysis revealed the existence of parameter spaces with two distinct causality structures: one characterized by a "negative spacelike signature" and the other by a "positive spacelike signature."

In the case of the "negative spacelike signature," we observed that the interval between two spacelike dendrograms was consistently negative (for n=6 and all n+k, k=1,2..6 levels). This negative signature held true across all intervals computed from the parameters of the 9 initial n=6 level dendrograms and their corresponding n+k spacelike dendrograms (where k ranged from 1 to 6). Notably, the timelike dendrograms derived from each of the n=6 initial dendrograms consistently exhibited positive values for their respective time-like parameter intervals. Importantly, this reversed causality structure was consistently observed across all 9 initial dendrograms and their unique sets of n+k dendrograms (totaling 21, 51, 127, 127, 834, and 2400 such unique dendrograms for k=1, 2, ..., 6, respectively).

This observation contradicts the ordinary causal structure of the real Minkowski metric in special relativity and its resulting characteristic light-cone , where information cannot be transmitted between two spacelike separated events for the trivial cause that the sum of the space coordinates' intervals is greater than the distance light can travel within the given time interval.

Conversely, the "positive spacelike signature" aligns with the characteristics of the Minkowski metric. this positive "space like signature" remained consistent for all intervals computed from the parameters of the 9 initial n=6 level dendrograms and their corresponding n+k spacelike dendrograms (with k ranging from 1 to 6). this Minkowski-like causality structure persisted across all 9 initial dendrograms and their unique sets of n+k dendrograms (totaling 21, 51, 127, 127, 834, and 2400 such dendrograms for k=1, 2, ..., 6, respectively).

In our random numerical analysis, we observed a higher occurrence of parameter spaces with a "positive spacelike signature" compared to those with a "negative spacelike signature." Remarkably, each of these two distinct parameter spaces maintained consistency and demonstrated opposite sign intervals between timelike and spacelike dendrograms across all initial n=6 dendrograms and their corresponding n+k level dendrograms.**Figure S3** illustrates the overall rather peculiar and counter-intuitive causality, where timelike intervals have positive values and spacelike intervals have negative values. **Figure S4** on the other hand illustrates the elignment of the "positive spacelike signature" with the regular Minkowski causal spacelike structure.

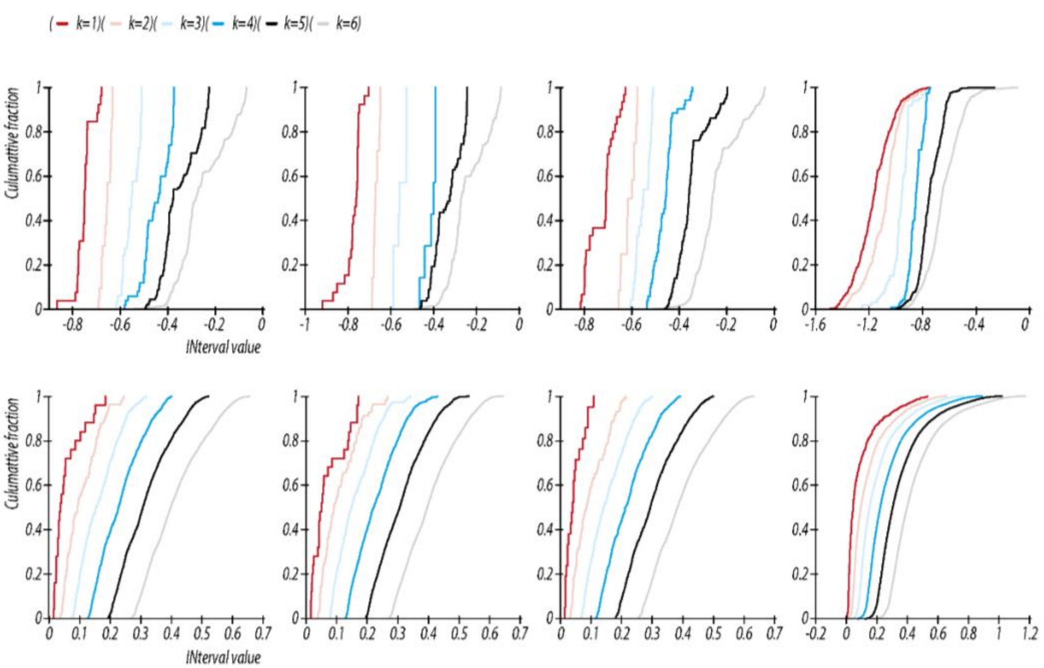

Figure S3: Time-Like and Spacelike Dendrogram Interval Values for the Negative Spacelike Signature Parameter Space. This figure explores the interval values for time-like and spacelike dendrograms in the context of the negative spacelike signature parameter space. The figure is divided into several panels, each representing different aspects of the analysis.**Top row first 3 panels from the left: Cumulative Distribution Functions (cdfs) of Interval Values for Spacelike Dendrograms** depict the cumulative distribution functions (cdfs) of INterval values for spacelike dendrograms, focusing on three specific initial dendrograms at the initial level n=6 and varying k values (k=1, 2, ..., 6). Each panel showcases the distribution of interval values for spacelike dendrograms resulting from the transformation and branching processes. **Top row panel on the right: Cumulative Distribution Function (cdfs) of Interval Values for All Spacelike Dendrograms** presents the cumulative distribution function (cdfs) of INterval values for spacelike dendrograms, encompassing all initial dendrograms at the initial level n=6 and for each k value (k=1, 2, ..., 6). The visualization offers an overall perspective of the interval value distributions across all spacelike dendrograms. **Bottom row first 3 panels from the left:**

Cumulative Distribution Functions (cdfs) of Interval Values for Time-Like Dendrograms illustrate the cumulative distribution functions (cdfs) of INterval values for time-like dendrograms, focusing on three specific initial dendrograms at the initial level n=6 and varying k values (k=1, 2, ..., 6). Each panel provides insights into the distribution of interval values for time-like dendrograms resulting from the transformation and branching processes. **Bottom rowrightpanel: Cumulative Distribution Function (cdfs) of Interval Values for All Time-Like Dendrograms** displays the cumulative distribution function (cdfs) of INterval values for time-like dendrograms, encompassing all initial dendrograms at the initial level n=6 and for each k value (k=1, 2, ..., 6). The visualization presents an overall perspective of the INterval value distributions across all time-like dendrograms.

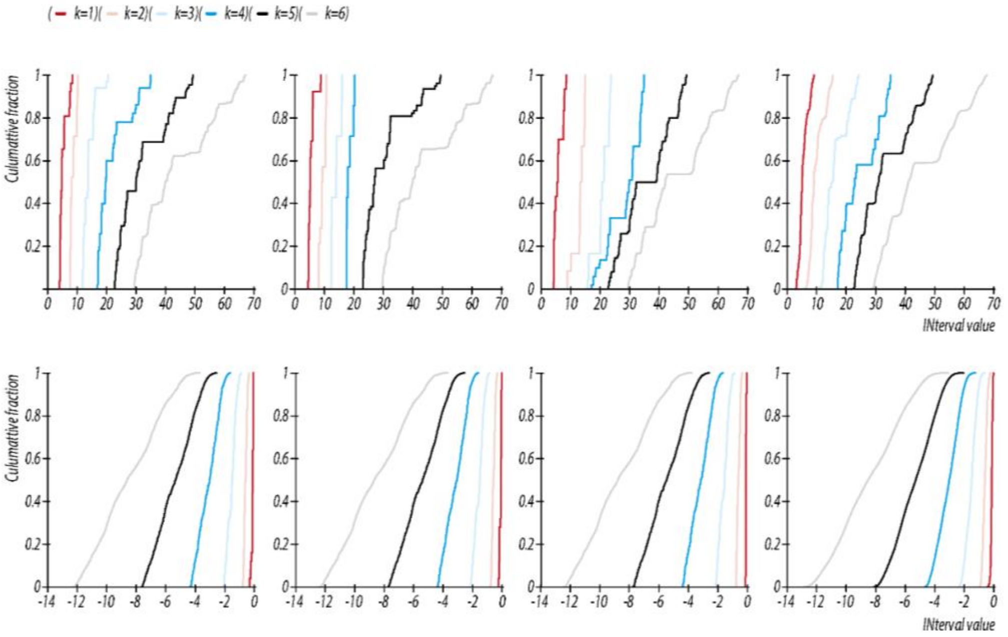

Figure S4: Time-Like and Spacelike Dendrogram Interval Values for the Positive Spacelike Signature Parameter Space This figure explores the interval values for time-like and spacelike dendrograms in the context of the positive spacelike signature parameter space. The figure is divided into several panels, each representing different aspects of the analysis.**Top row first 3 panels from the left: Cumulative Distribution Functions (cdfs) of Interval Values for Spacelike Dendrograms** depict the cumulative distribution functions (cdfs) of INterval values for spacelike dendrograms, focusing on three specific initial dendrograms at the initial level n=6 and varying k values (k=1, 2, ..., 6). Each panel showcases the distribution of interval values for spacelike dendrograms resulting from the transformation and branching processes. **Top row panel on the right: Cumulative Distribution Function (cdfs) of Interval Values for All Spacelike Dendrograms** presents the cumulative distribution function (cdfs) of INterval values for spacelike dendrograms, encompassing all initial dendrograms at the initial level n=6 and for each k value (k=1, 2, ..., 6). The visualization offers an overall perspective of the interval value distributions across all spacelike dendrograms.**Bottom row first 3 panels from the left: Cumulative Distribution Functions (cdfs) of Interval Values for Time-Like Dendrograms** illustrate the cumulative distribution functions (cdfs) of INterval values for time-like dendrograms, focusing on three specific initial dendrograms at the initial level n=6 and varying k values (k=1, 2, ..., 6). Each panel provides insights into the distribution of interval values for time-like dendrograms resulting from the transformation and branching processes. **Bottom row panel on the right: Cumulative Distribution Function (cdfs) of Interval Values for All Time-Like Dendrograms** displays the cumulative distribution function (cdfs) of INterval values for time-like dendrograms, encompassing all initial dendrograms at the initial level n=6 and for each k value (k=1, 2, ..., 6). The visualization presents an overall perspective of the INterval value distributions across all time-like dendrograms.

Out of the 5000 randomly generated combinations of constants (z, z1, z2, z3, z4, and s2), our analysis identified 260 distinct combinations that demonstrated consistency across all n+k levels for the initial set of 9 dendrograms with n=6. This consistency was defined by the inverse sign between the computed $INterval$ values, as defined in equation 6, for the timelike dendrograms and their corresponding spacelike dendrograms.

In total, we discovered a total of 3117 different combinations of constants (z, z1, z2, z3, z4, s2) and parameters (θ'_1, θ'_2, θ'_3, θ'_4) that exhibited this remarkable consistency. Among these combinations, 797 displayed a "negative spacelike signature," while 2320 exhibited a "positive spacelike signature" (as depicted in Figure **S5**).

We then verified the consistency of the found sets of these 3117 parameters and constants in the same way for the initial dendrograms of n=5 and n=7.

We observed that when preparing the dendrogram parameters according to the above described method, only specific combinations of $\theta'_1, \theta'_2, \theta'_3\ and\ \theta'_4$ can uniquely describe a dendrogram. Such uniqueness is achieved when one of the parameters ($\theta'_1, \theta'_2\ \ or\ \theta'_3$) is either V_D or U_D, and another parameter is a product, sum, or division of the two. Alternatively, when one of the parameters is R_D or r_D, and another parameter is a product, sum, or division of the two.

Figure S5: number of "negative spacelike signature and positive spacelike signature parameter spaces found in our simulations

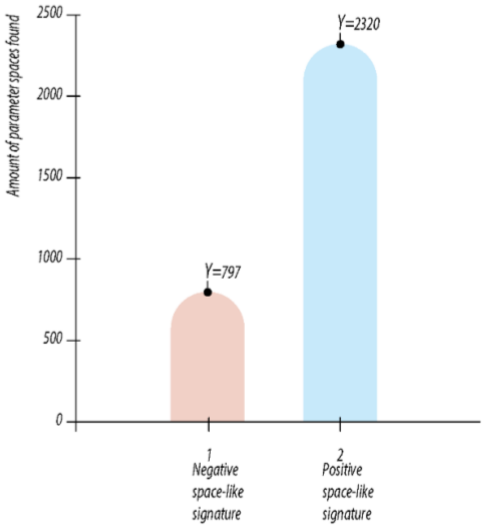

Interestingly, we discovered that all 797 combinations with a "negative spacelike signature" were unable to uniquely define the dendrogram space. This suggests that in order for a coordinate/parameter space to uniquely define events (or dendrograms in our case), as is the case in ordinary spacetime, it must possess the Minkowski light-cone characteristics and vice versa. In these cases with "negative spacelike signature," where this condition was reversed, we lost the uniqueness definition of dendrograms.

Out of the total 2320 consistent parameters that exhibited a "positive spacelike signature," only 310 combinations were found to uniquely define the dendrogram space (refer to Figure **S6**).

To further investigate this conjecture, we examined a mixture of 760*137 parameter combinations that possessed either a "negative spacelike signature" or a "positive spacelike signature" in n=6 while still being able to uniquely define the dendrogram space.

As expected, all the parameters with a "negative spacelike signature" could not exhibit consistent opposite signs for all m unique initial dendrograms with n=5,7 and their corresponding n+k level spacelike/timelike dendrograms where k=1,2..6. Consequently, these parameters failed to maintain the causal structure of the “reversed” non-trivial light-cone for all n’s.

Thus in cases of parameters exhibiting consistent "negative spacelike signature" with “reversed” causal structure and non-trivial light-cone they fail to uniquely define the dendrogram space. while in cases that do possess the ability to uniquely define the dendrograms space the "negative spacelike signature" loose consistency of this reversed causal structure.

Figure S6: number of positive spacelike signature that uniquely define the dendrogram space vs positive spacelike signature that did not uniquely define the dendrogram space found in our simulations.

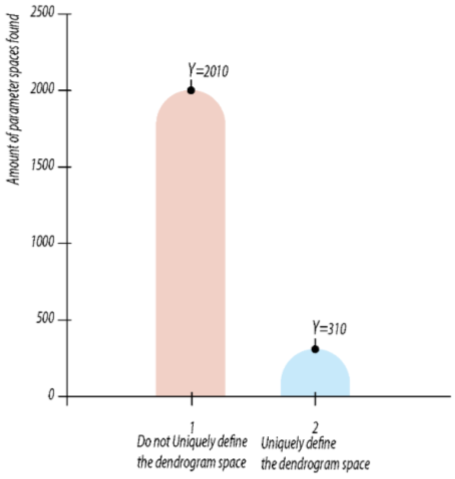

On the other hand, some of the parameter spaces (49 of them) with a "positive spacelike signature" exhibited the necessary consistency of the causal structure of the light cone, in all tests conducted for n=5, 6, and 7 (as described earlier). This outcome confirmed that the only possible causal structure must resemble the ordinary Minkowski metric light cone.

In order to validate the applicability of the 310 combinations of $\theta'_1, \theta'_2, \theta'_3$ and θ'_4 that were both consistent and could uniquely define the dendrogram space we conducted the following test: 20000 times we randomly generated a dendrogram with a random size smaller than 100 events and its corresponding timelike dendrogram, which was obtained by adding random k events to the initial dendrogram. The value of k satisfied the condition;
*number of events in initial dendrogram<k<2*number of events in initial dendrogram*
Among the 20,000 tests conducted on “positive space-like signature spaces” none of the cases yielded positive values, thereby illustrating the consistency of the time-like negative signature. This finding is exemplified in Figure S7. One such of the parameter spaces that showed consistency in all m dendrograms for all n=5,6,7 and k=1,2…6 proved to uniquely define the dendrogram space already in section 3.

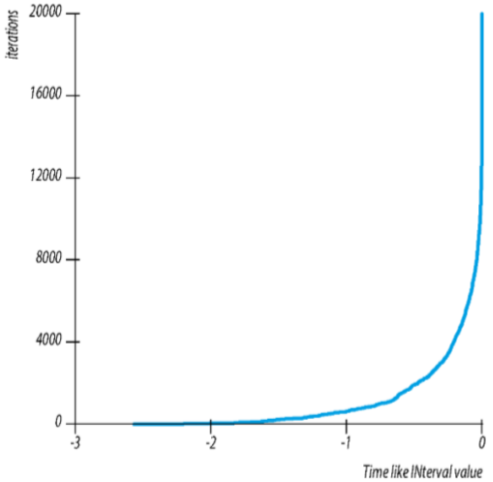

Figure S7: Illustration of the consistency of one time-like negative signature parameter space found in our simulations. This parameter space uniquely defined the dendrogramic space .

A2.1 The observer subjective wave function

We start with constructions of the “differences pdf”, ρ. This will be our fundamental distribution. Thus, given a dendrogram that describes, p-adicaly, the relations between m events we calculate all possible pairwise differences of the p-adic edges representation and represent them as events through monna map as above in equations 1.1-1.3:

$$q_{ik} = (event_i - event_k)$$

Where for p = 2,
$edge_i = \sum_{j=0}^{k} a_j \times p^j \quad \rightarrow \; event_i = \sum_{j=0}^{k} a_j \times p^{-j-1}, \; a_j = 1,0,$ the Monna map maps natural numbers into rational numbers belonging the segment [0,1]. A dendrogram is mapped into a subset of [0,1]. This map can be extended to the infinite p-adic tree where its branches are represented by infinite series; for p=2,
$edge_i = \sum_{j=0}^{\infty} a_j \times 2^j \quad \rightarrow \; event_i = \sum_{j=0}^{\infty} a_j \times 2^{-j-1}, \; a_j = 1,0,$ as in (2.1)
The latter is important for considering the subjective knowledge of an observer in the limit infinitely many events collected by observations; they are portrayed on an infinite 2-adic tree.
Thus, from all q_{ik} we have a discrete “difference pdf”, ρ, which is the fraction of each unique value of q_{ik} defined For the set of the unique q_{ik} values Q we have:

$$\rho_j = \frac{(number\ of\ different\ q_{ik}\ \ that\ equal\ Q_j)}{total\ number\ of\ q_{ik}} \qquad (2.2)$$

We then define the differences energy, which will be equivalent to the usual kinetic energy, we define the equation:
$p = \frac{1}{N}\sum_{k \neq j} q_{jk}$ in which N is the number of possible q_{jk}. (2.3)
This formula represents the mean difference between all pairwise events. Thus, p takes the part as the momentum in Bohmian mechanics.
Now, we introduce the phase S as the new parameter of the model:
We set $p = \partial S$ and $\partial S = e^{iS}$. (2.4)
We can then calculate the “differences energy” as the real part of:

$$T_{differences\ energy} = \frac{1}{N}\sum_{j \neq k}(q_{jk})^2 \qquad (2.5)$$

Where $T_{differences\ energy}$ serves as our equivalent of kinetical energy
The continuum approximation of $T_{differences\ energy}$ will be obtained by noticing

$$p_j = \rho(Q_j)(Q_j)^2 = \partial S_j \qquad (2.6)$$

Thus:

$$T_{differences\ energy} = \int (\rho(Q_j)(Q_j)^2)\rho(Q_j)dQ_j = \int (\partial S_j)^2 \rho(Q_j)dQ_j \quad (2.7)$$

By using the proceedures outlined in [5,43] and[43] we derive the action principle:

$$A(S,\rho) = \int d(Dendrogram)\{\int \frac{d(S)}{dDendrogram}\rho(Q)dQ + \int (\partial S)^2\rho(Q)dQ - \mathrm{v}(Q) + U(Q)\}$$

But from the results obtained in section 2 we can rewrite the action:

$$A(S,\rho) = \int d(\boldsymbol{\theta})\{\int \frac{d(S)}{d\boldsymbol{\theta}}\rho(Q)dQ + \int (\partial S)^2\rho(Q)dQ - \mathrm{v}(Q) + U(Q)\}$$

Where $\mathrm{v}(Q) = Z_V \int \rho(Q)(\frac{(\partial\rho(z))^2)}{(\rho(Q))^2})\, dQ$ and $U(Q) = \int \rho(Q)dQ$

(2.8)

Where $U(Q) = \int \rho(Q)dQ$

The Hamilton Jacobi equation of the action (notice we are on the straight line [0 1] now in all our pdfs).

$$-\dot{S} = (\partial_{edge}S)^2 + U + U^Q \quad (2.9)$$

In which $U^Q = (\Delta^2\sqrt{\rho})/\sqrt{\rho}$ is the quantum potential

And $U\ is$ the potential

And $\dot{S} = S(present\ dendrogram) - S(previous\ dendrogram)$

All parameters are well-defined. Thus,The probability conservation law is

$$\dot{\rho} = \partial_{edge}\ (\rho\partial_{edge}S)^2$$

In which $\dot{\rho} = \rho(present\ dendrogram) - \rho(previous\ dendrogram)$ (2.10)

Equations 10 and 11 are the real and imaginary parts of the Schrodinger equation for $\psi_{subjectve} = \sqrt{\rho}e^{iS}$.

We emphasize that the subjective wave function, $\psi_{subjective}$, is completely dependent on the measurements the observer is preforming thus for a set *M={m₁,m₂.. mᵢ}* ,

$\psi_{subjective}(M) = \psi_{subjective}(\boldsymbol{\theta})$ where different sets M can have same $\boldsymbol{\theta}$.

an observer may be characterized uniquely by its infinite set of measurements he preforms. In that sense these infinite set of measurements are its world line curve (accelerated or not) on the background of spacetime ($\boldsymbol{\theta}$), each observer will ultimately obtain different ontic relational view of the universe (two observers with same world line are not allowed as they are the same observer if we postulate Leibnitz principle). This view is dependent on the information about events, localized in the background of spacetime, transmitted to the observer moving with acceleration or without it in spacetime. The transition of this kind of measurements "world line" into the dynamical evolvement of the subjective wave function, $\psi_{subjective}(\boldsymbol{\theta})$, is trivial.

A2.2 Coupling to Rovelli's relational quantum mechanics

It's important to highlight that DHT does not fall within the confines of either the quantum or classical paradigms. Both these paradigms naturally emerge from the p-adic relational tree, as demonstrated in [1–3,49], without the need for any additional assumptions other than the acceptance of the Leibniz Principle.

Notably, *Rovelli's Relational Quantum Mechanics* (RQM) shares a significant ideological similarity with DHT [50]. However, RQM posits that all systems are inherently quantum systems. Like DHT, RQM leverages the concept that any quantum mechanical measurement can be deconstructed into a series of yes-no questions, which is then used to formulate the state of a quantum system (relative to a given observer, much like in DHT).

In contrast to DHT, RQM asserts the completeness of quantum mechanics. Accordingly, RQM posits that there are no hidden variables or additional factors that need to be introduced into quantum mechanics, based on current experimental evidence. As demonstrated in [5], quantum theory can be viewed as an emerging theory stemming from a relational structure. Consequently, notions such as completeness and hidden variables become irrelevant. From this perspective, the various interpretations of quantum mechanics can be seen as corresponding to different emergence frameworks for quantum theory from an event-based relational structure

Recently more postulates where added to the RQM interpretation [51] :

1. Relative facts: Events, or facts, can happen relative to any physical system.

In our model framework the subjectivity of information acquired by an observer fullfils this postulate more over $\psi^{O_{B_k}}$ which is the objective wave function, or property of the observer is only a relative concept to other observer.

2. No hidden variables: Unitary quantum mechanics is complete.
As demonstrated in [5] and in the current model, quantum theory can be seen as emerging from a relational structure. Consequently, concepts like completeness and hidden variables become irrelevant.
3. Relations are intrinsic: The relation between any two systems A and B is independent of anything that happens outside these systems' perspectives.
As is shown in the current model interaction/measurments between observers or group of observers pretaines only to the relational information one observer (group of observers) acquire on the other (other group). In that sense also the "objective" $\psi^{O_{B_k}}$ is dynamically evolving only in relation to another observer or group of observers.

4. Relativity of comparisons: It is meaningless to compare the accounts relative to any two systems except by invoking a third system relative to which the comparison is made.
In our model, each observer possesses a description of the scenario that is accurate from their perspective. However, due to the relativity of comparisons, these descriptions cannot be meaningfully compared. This parallels the situation described in Wigner's friend case, as elucidated by Rovelli regarding the significance of the aforementioned postulate.

5. Measurement: An interaction between two systems results in a correlation within the interactions between these two systems and a third one; that is, with respect to a third system W, the interaction between the two systems S and F is described by a unitary evolution that potentially entangles the quantum states of S and F.
In our model observer S and F are measured by W their eignfunctions are both now part of its world line and evolve in full correlation to the worldline trajectory of W

6. Internally consistent descriptions: In a scenario where F measures S, and W also measures S in the same basis, and W then interacts with F to "check the reading" of a pointer variable (i.e., by measuring F in the appropriate "pointer basis"), the two values found are in agreement.
In our model, two "same readings" correspond to the same eigenfunctions in the observer's world line, which results in no movement of the observer through the **θ** parameter space upon the second checking. Consequently, interaction without movement leads to the same pointer.

Please notice again that all postulates of RQM are emergent in the relational information framework.

A2.3 Coupling to Smolin's approach of emergence of quantum mechanics
It's worth mentioning Smolin's exploration of the emergence of quantum mechanics and spacetime, which introduces rationalism perspectives, as evident in works such as energetic causal set theory and [43–46]. While these works share a similar ideology with DHT, they are constructed based on concepts like momentum, energy, and even coordinates. Interestingly, these elements are entirely absent in the foundational construction of DHT, yet they can still potentially emerge from the relational p-adic structure. This is the important foundational advantage of DHT: space-time is not considered as the basic concept, but it is derived from treelike structure of events collected by an observer. In this way, spacetime is subjective – observer dependent (as described in section 2) [3,6]
Additionally, we'd like to highlight certain parallels between our approach, which leads to the emergence of quantum theory from DHT, and the neural network model of the universe as developed in articles [38–42]

A3.1
For concreteness, we consider $p = 2$.

Step 1. From a data time series to a dendrogram: a hierarchic clustering of data.
time series of data, $Z1, Z2, \dots, Zn$. Split it into blocks of length d. from each block constructed (with a hierarchic clustering algorithm) a dendrogram with d edge nodes resulting in a dendrogramic time series, $D_1, D_2, \dots, D_s$.
Step 2. From a dendrogram time series to a 2-adic time series.
each dendrogram D can be represented by d vectors $(\alpha 0, \alpha 1, \dots, \alpha k)$, where $\alpha j = 0,1$. Each vector encodes root to end node path on D transform the vector representation to natural numbers representations, by using the following formula:

$$(\alpha_0\alpha_1\ldots\alpha_k) = \alpha_0 + \alpha_1 2 + \alpha_2 2^2 + \ldots + \alpha_k 2^k.$$

these natural numbers were sorted in ascending order in each block of length d for each dendrogram D_n.
Thes natural numbers are analogs of polarization in the real CHSH experiment.

for correlations, we considered two time series, $Z1, Z2, \dots, Zn$ and $Z1', Z2', \dots, Zn'$. Applyi the procedures of Steps 1 and 2, we obtain two series of natural numbers. They are formed from blocks of d natural numbers, which were generated through the d-decomposition of the original time series:

$x = x_1, x_2, \dots, x_n$, *x_n is composed of blocks with d natural numbers*
$y = y_1, y_2, \dots, y_n$, *y_n is composed of blocks with d natural numbers*

Step 3. Defining the observables.

First method

We construct two time series of dendrograms, one for **Alice A = (A1, A2, A3, . . . , An)** and one for **Bob B =(B1,B2,B3,.. ,Bn)**. For **Alice,** we. Select two pairs of numbers, **a** = [a1 a2] and **a’** = [a1′ a2′]. The two pairs are not identical. The analogs of the two vectors are orientations of polarization beam splitters or Stern–Gerlach magnets.

For **Bob,** we select two pairs of numbers, **b** = [b1 b2] and **b’** = [b1′ b2′]. The two pairs are not identical. For each Ai i∈1, 2, 3, . . . , n, randomly decide between pair a or a’. For each Bi i∈1, 2, 3, . . . , n, randomly decide between pair b or b’. If, for **A**i, pair **a** is chosen**,** then:

If **A**i had both of the numbers in **a,** Sai = 1, otherwise, Sai= −1.

Metaphorically, we can say that if the “polarization” of **A**i coincides with a, the detector with the output is +1 clicks, if not, the detector with the output is −1 clicks.

proceed the same for a selection of a’ and b, b’. calculate the correlations, as follows: Cab = (∑Sai*Sbi)/length(a and b are selected together) Cab’ = (∑Sai*Sb’i)/length(a and b’ are selected together) Ca’b = (∑Sa’i*Sbi)/length(a’ and b are selected together) Ca’b’ = (∑Sa’i*Sb’i)/length(a’ and b’ are selected together) C= Cab − Cab’ + Ca’b + Ca’b’

Second method

constructed two time series of dendrograms, one for **Alice A = (A1, A2, A3, . . . , An)** and one for **Bob B=(B1,B2,B3,... ,Bn)**.

For **Alice,** select two nonidentical pairs of numbers, **a** = [a1 a2] and **a’** = [a1′ a2′].

For **Bob** we select two nonidentical pairs of numbers **b**=[b1 b2] and **b’**=[b1’ b2’] .

For each Ai i∈1, 2, 3, . . . , n, randomly decide between pair a or a’. For each Bi i∈1, 2, 3, . . . , n, randomly decide between pair b or b’. If for **A**i pair **a** is chosen**,** then:

replace the natural number in Ai that equal a1 to 1
replace the natural number in Ai that equal a2 to −1
replace all other natural numbers in Ai to zero. indicate that pair **a** was chosen to Ai.
The same we do for other settings, a’, b, b’-
find i that a and b were chosen = nab,
find i that a’ and b were chosen = na’b,
find i that a and b’ were chosen = nab’,
find i that a and b’ were chosen = na’b’,
calculate Sab = (Ai = nab) *(Bi = nab).
calculate Sa’b = (Ai = na’b) *(Bi = na’b).
calculate Sab’ = (Ai = nab’) *(Bi = nab’).
calculate Sa’b’ = (Ai = na’b’) *(Bi = na’b’).

For block j of size d of Sab, if there are two values of 1, Xabj = 1, if there is one value of 1, Xabj = 1, if there is one value of −1 Xabj = −1, if all values are 0, Xabj = −1.

For block j of size d of Sab, if there are two values of 1, Xa’bj = 1, if there is one value of 1, Xa’bj = 1, if there is one value of −1, Xa’bj = −1, if all values are 0, Xa’bj = −1.

For block j of size d of Sab, if there are two values of 1, Xab’j = 1, if there is one value of 1, Xab’j = 1, if there is one value of −1, Xab’j = −1, if all values are 0, Xab’j = −1.

For block j of size d of Sab, if there are two values of 1, Xabj = 1, if there is one value of 1, Xa’b’j = 1, if there is one value of −1, Xa’b’j = −1, if all values are 0, Xa’b’j = −1.

calculate:

Cab = (∑Xabj)/length(Xabj) Cab’ = (∑Xab’j)/length(Xab’j) Ca’b = (∑ Xa’bj)/length(Xa’bj) Ca’b’ = (∑ Xa’b’j)/length(Xa’b’j) C = Cab-Cab’ + Ca’b + Ca’b’
